# Effects of Single Nucleotide Polymorphisms in Human *KCNMA1* on BK Current Properties

**DOI:** 10.3389/fnmol.2019.00285

**Published:** 2019-12-03

**Authors:** Amber E. Plante, Michael H. Lai, Jessica Lu, Andrea L. Meredith

**Affiliations:** Department of Physiology, University of Maryland School of Medicine, Baltimore, MD, United States

**Keywords:** BK channel, KCa1.1, calcium-activated potassium channel, *KCNMA1*, potassium channel, MaxiK, *Slo*, *slopoke*

## Abstract

BK Ca^2+^-activated K^+^ channels are important regulators of membrane excitability. Multiple regulatory mechanisms tailor BK current properties across tissues, such as alternative splicing, posttranslational modifications, and auxiliary subunits. Another potential mechanism for modulating BK channel activity is genetic variation due to single nucleotide polymorphisms (SNPs). The gene encoding the human BK α subunit, *KCNMA1*, contains hundreds of SNPs. However, the variation in BK channel activity due to SNPs is not well studied. Here, we screened the effects of four SNPs (A138V, C495G, N599D, and R800W) on BK currents in HEK293T cells, selected based on predicted protein pathogenicity or disease linkage. We found that the SNPs C495G and R800W had the largest effects on BK currents, affecting the conductance–voltage relationship across multiple Ca^2+^ conditions in the context of two BK channel splice variants. In symmetrical K^+^, C495G shifted the V_1/2_ to more hyperpolarized potentials (by −15 to −20 mV) and accelerated activation, indicating C495G confers some gain-of-function properties. R800W shifted the V_1/2_ to more depolarized potentials (+15 to +35 mV) and slowed activation, conferring loss-of-function properties. Moreover, the C495G and R800W effects on current properties were found to persist with posttranslational modifications. In contrast, A138V and N599D had smaller and more variable effects on current properties. Neither application of alkaline phosphatase to patches, which results in increased BK channel activity attributed to channel dephosphorylation, nor bidirectional redox modulations completely abrogated SNP effects on BK currents. Lastly, in physiological K^+^, C495G increased the amplitude of action potential (AP)-evoked BK currents, while R800W had a more limited effect. However, the introduction of R800W in parallel with the epilepsy-linked mutation D434G (D434G/R800W) decreased the amplitude of AP-evoked BK currents compared with D434G alone. These results suggest that in a physiological context, C495G could increase BK activation, while the effects of the loss-of-function SNP R800W could oppose the gain-of-function effects of an epilepsy-linked mutation. Together, these results implicate naturally occurring human genetic variation as a potential modifier of BK channel activity across a variety of conditions.

## Introduction

BK channels are large-conductance voltage- and Ca^2+^-activated K^+^ channels encoded by a single gene product (*KCNMA1* in humans or *Slo1* in mouse; Atkinson et al., 1991; Adelman et al., 1992; Butler et al., 1993). The pore-forming BK channel is a tetramer of α subunits (Shen et al., 1994; Meera et al., 1997). Each α subunit consists of a membrane-spanning domain that forms the channel pore and voltage-sensing domains (Meera et al., 1997; Stefani et al., 1997; Ma et al., 2006) and a cytosolic domain that contains two regulators of K^+^ conductance (RCK) domains that mediate Ca^2+^-dependent gating (Magleby, 2003; Salkoff et al., 2006). In most excitable cells, voltage and Ca^2+^ act in concert to activate BK channels (Latorre et al., 2017).

BK channels are widely expressed throughout the body. Therefore, intrinsic BK channel properties are regulated in a tissue-specific manner by co-expressed auxiliary beta (β) subunits (β1–β4) or gamma (γ) subunits (γ1–γ4; Li and Yan, 2016; Gonzalez-Perez and Lingle, 2019), alternative splicing (Shipston, 2001; Glauser et al., 2011), and posttranslational modifications (Kyle and Braun, 2014). These regulatory mechanisms allow functional diversity of BK current properties that is crucial to the wide-ranging roles that BK channels play within the body (Latorre et al., 2017). Because BK currents mediate physiological functions critical to human health, such as vascular and cardiac muscle function, heart rate, bladder function, and circadian rhythm (Nelson et al., 1995; Meredith et al., 2004, 2006; Werner et al., 2005; Lai et al., 2014), identifying sequence variations within the human *KCNMA1* gene that alter BK channel properties may lend insight into the development of pathophysiology and disease in humans.

Rare genetic variations, in the form of mutations, in genes encoding human ion channels are linked to more than 100 “channelopathies” (Meredith, 2015). A plethora of BK channel mutations conferring gain-of-function (Du et al., 2005; Zhang et al., 2015; Li et al., 2018) and loss-of-function properties (Carvalho-de-Souza et al., 2016; Staisch et al., 2016; Tabarki et al., 2016; Yeşil et al., 2018; Liang et al., 2019) have been identified from human patients (Bailey et al., 2019). One well-characterized mutation in the BK channel α subunit, which leads to the D434G substitution in the RCK1 domain, conveys gain-of-function properties to BK currents (Yang et al., 2010) and has been shown as causative for epilepsy and paroxysmal dyskinesia (Du et al., 2005). It has been proposed that this gain-of-function D434G mutation causes faster repolarization of action potentials (APs), leading to an increase in neuronal excitability (Wang et al., 2009).

In contrast to mutations, single nucleotide polymorphisms (SNPs) are the most common source of genetic variation and account for the majority of differences between individuals (Frazer et al., 2007). SNPs consist of a single base pair change every ~1,000 nucleotides, and there are ~4–5 million SNPs in a single human genome (Sachidanandam et al., 2001; Auton et al., 2015). SNPs are heritable and, in some cases, are linked to disease susceptibility as well as neurological and cardiovascular disorders (Meredith, 2015). SNPs are typically classified as mutations when genetic inheritance of a rare substitution is linked to a pathological disorder.

Several SNPs in the genes that encode the BK α subunit (*KCNMA1*) and auxiliary β subunits (*KCNMB1–4*) have also been linked to human disorders including autism (Laumonnier et al., 2006), cardiovascular function (Gollasch et al., 2002; Köhler, 2010), and asthma (Seibold et al., 2008; Valverde et al., 2011). SNPs in the gene encoding the auxiliary β1 subunit, *KCNMB1*, are associated with increased variability in heart rate and baroreflex function and sex-specific asthma susceptibility (Gollasch et al., 2002; Seibold et al., 2008). One SNP, A138V, in the intracellular S0–S1 linker of the BK channel α subunit, was associated with autism in a patient carrying a 10q22 chromosomal translocation (Laumonnier et al., 2006). BK currents evoked from a cell line derived from this individual were found to be reduced by 70% compared with control patients. These studies suggest that alterations in BK currents induced by SNPs could have pathophysiological implications. However, the vast majority of the non-synonymous SNPs that alter the coding sequence of the BK channel membrane-spanning region and cytosolic gating ring domain are not yet linked to disease risk. Therefore, characterization of additional SNPs in *KCNMA1* may uncover further variation in BK channel properties that have the potential to influence physiological function.

**Figure 1:**
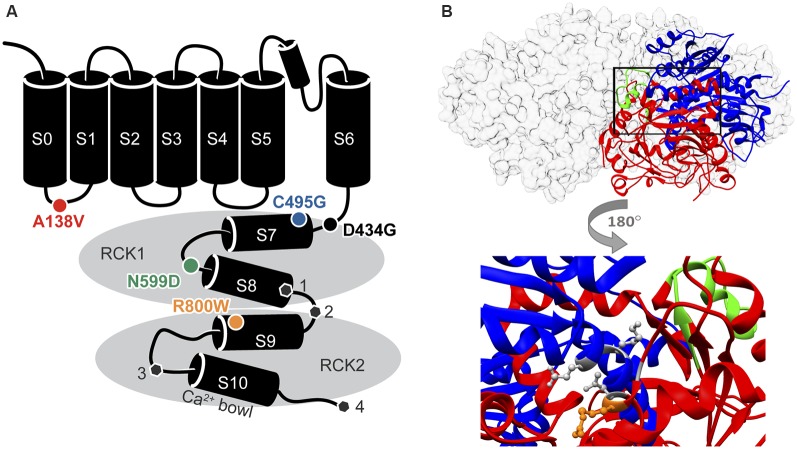
Locations of single nucleotide polymorphism (SNP) residues in the BK channel α subunit. **(A)** SNPs located in the in the S0-S1 linker (A138V) and the intracellular “gating ring” domain (C495G, N599D, and R800W) were introduced into two human BK channel splice variants. The sites of alternative splicing (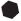, sites 1–4) differing between hBK_VYR_ and hBK_QEERL_ channel sequences (gray hexagons). **(B)** *Top*, side view of the gating ring structure surface structure (gray) with one subunit as a ribbon diagram showing the RCK1 (blue) and RCK2 (red) domains and the Ca^2+^ Bowl (green; PDB 3NAF; Wu et al., 2010). *Bottom*, magnified portion of the ribbon diagram from above rotated 180 degrees to show the location of residue R800 (orange stick) in relation to residues G798 and N801 in RCK2, which interact with K555 and E553 in RCK1, respectively, to make up part of the RCK1–RCK2 “flexible interface” (gray sticks; Kim et al., 2008).

To test whether additional non-synonymous SNPs in *KCNMA1* may contribute to BK current diversity, structural information and computational prediction algorithms were initially used to hypothesize which SNPs had a strong potential to affect protein function. Using these algorithms in combination with prior structure–function studies, we identified four high priority SNPs in the BK α subunit (Table 1 and Figure 1A). We tested the effects of these SNPs on channel function in a heterologous expression system by recording BK currents properties from SNP-containing channels in symmetrical K^+^ under a range of Ca^2+^ concentrations and in physiological solutions with neuron and muscle-derived AP commands. We found two SNPs, C495G and R800W, had relatively consistent effects on BK current properties under a variety of conditions. These SNPs were subjected to additional mutagenesis experiments to explore the mechanism of the SNP-induced changes in BK channel function.

**Table 1:** Single nucleotide polymorphisms (SNPs) predicted to modulate human BK channel properties.

SNP ID	Substitution	MutPred2 scores		Description of chosen SNPs
		hBK_QEERL_	hBK_VYR_	
rs144215383	A138V	0.089 (low)	0.084 (low)	SNP isolated from autistic patients (Laumonnier et al., 2006) Cells from an autistic patient exhibit reduced BK current and depolarized resting membrane potential compared with controls (Laumonnier et al., 2006) SNP located near Mg^2+^ coordination residues (Yang et al., 2008)
rs201243440	C495G	0.815 (high)	0.818 (high)	C495 mediates BK channel oxidation and current rundown (Zhang et al., 2006) Mutation of C495 inhibits BK current modulation by cysteine-modifying reagents (Zhang and Horrigan, 2005) SNP adds a flexible glycine residue into a conserved linker region in RCK1 (Jiang et al., 2001; Zhang and Horrigan, 2005)
rs140520584	N599D	0.654 (medium)	0.668 (medium)	Mutation of N599 alters Ca^2+^-dependent channel activation (Zhang et al., 2010) SNP introduces a negative charge adjacent to E600, a residue critical for Ca^2+^ sensing in RCK1 (Zhang et al., 2010)
rs199681253	R800W	0.768 (high)	0.754 (high)	Located adjacent G798 and N801, residues implicated in regulating the flexible interface between RCK1 and RCK2 (Kim et al., 2008) SNP introduces a large bulky residue, eliminates a positive charge and potential methylation site

*Background and MutPred2 scores for each SNP substitution introduced into channel hBK_QEERL_ (GenBank MG279688) and hBK_VYR_ (GenBank MG279689) protein sequences. Scores range from 0 (low probability) to 1 (high probability) for alteration of protein function. SNPs were identified from the National Center for Biotechnology Information dbSNP database*.

## Materials and Methods

### Identification of Single Nucleotide Polymorphisms in *KCNMA1*

Over 150 SNPs in the human *KCNMA1* gene encoding the BK channel α subunit (gene ID: 3778; 10q22.3) were identified from the National Center for Biotechnology Information dbSNP repository^1^ (Sherry et al., 2001). Of these, 99 non-synonymous SNPs were introduced into the BK channel complementary DNA sequences for hBK_QEERL_ (GenBank MG279688) and hBK_VYR_ (GenBank MG279689) splice variants and analyzed *in silico* with the MutPred2 web application^2^ (Li et al., 2009; Pejaver et al., 2017; Table 1). These sequences included a Myc and EYFP tag. MutPred2 scores were similar for hBK sequences with and without the Myc and EYFP tags (data not shown). All sequence numbering in the text refers to sequence positions in the untagged hBK_QEERL_ background. The human BK channel gating ring structure (PDB: 3NAF; Wu et al., 2010) in Figure 1 was generated using University of California, San Francisco Chimera (Pettersen et al., 2004).

### Synthesis of Single Nucleotide Polymorphism-Containing Human BK Channel Variants

The human BK channel (hBK) complementary DNA was generated by replacing eight amino acids in the mouse clone (mBK_VYR_; GenBank JX462786) with the human residues (hBK_VYR_; GenBank MG279689). The hBK_QEERL_ variant was generated by deleting the “SRKR” exon from splice site 1 and the Ca^2+^ bowl exon from site 3 within a *PciI* fragment (4, 981 bp). The human “QEERL” exon at site 4 was introduced by subcloning the *PciI* fragment into a *PciI*-digested mouse BK_QEERL_ clone (GenBank KF530043), resulting in the final construct called hBK_QEERL_ (GenBank MG279688). hBK_VYR_ and hBK_QEERL_ were sequenced in entirety and verified to have no extraneous mutations. SNP sequences or mutations were introduced into hBK_QEERL_ and hBK_VYR_ backgrounds by site-directed mutagenesis (Bioinnovatise, Rockville, MD, USA) in the pcDNA3.1+ mammalian expression vector and verified by sequencing. All channel constructs contain an N-terminal Myc tag and an EYFP tag (241 amino acids) inserted after residue 742 in the RCK2 domain.

### Cell Culture and Electrophysiology

HEK293T cells (CRL-11268, ATCC, Manassas, VA, USA) were cultured in a 37°C incubator with 5% carbon dioxide in 35- and 60-mm tissue culture dishes in media containing Dulbecco’s modified Eagle medium (cat. #11995-065, Gibco, Life Technologies Corp., Grand Island, NY, USA), 10% fetal bovine serum (cat. #4135, Sigma-Aldrich, St. Louis, MO, USA), 1% penicillin/streptomycin (cat. #30-002-Cl, Mediatech Inc., Manassas, VA, USA), and 1% L-glutamine (cat. #25-005-Cl, Mediatech Inc., Manassas, VA, USA). Cells were transfected at 50–70% confluency with wild-type (WT) or SNP-containing BK expression constructs using either Lipofectamine 2000 (Life Technologies Corp., Grand Island, NY, USA) or Trans-IT LT1 (Mirius Biological, Madison, WI, USA) at 1/5 and 1/2 ratios of DNA/transfection reagent (μg/μl), respectively, according to manufacturer protocols. After 6–12 h, cells were re-plated onto glass coverslips pre-coated with poly-L-lysine (cat. #P4832, Sigma-Aldrich, St. Louis, MO, USA) and recorded from 20 h to 48 h post-transfection. BK-expressing cells were identified by the fluorescence signal from the EYFP tag in the BK α subunit. No differences in expression were observed between WT and any of the SNP-containing channels.

Inside out patch clamp recordings of macroscopic BK currents were conducted at room temperature using thin-walled borosilicate glass pipettes with resistances of 1–4 MΩ (cat. #TW150F-4, World Precision Instruments, Sarasota, FL, USA). Data were acquired at 50 kHz and online filtered at 10 kHz with the MultiClamp 700B amplifier (Axon Instruments, Sunnyvale, CA, USA). For symmetrical K^+^ experiments, the external (pipette) solution contained (mM): 140 KMeSO_3_, 2 KCl, 2 MgCl_2_, and 20 HEPES. Internal (bath) solution contained (in mM): 140 KMeSO_3_, 2 KCl, and 20 HEPES. pH was adjusted to 7.2 with KOH. Appropriate amounts of CaCl_2_, calculated in WebMaxC^3^, were added to the internal solution to achieve the indicated concentrations of free Ca^2+^. Solutions were buffered with either 5-mM EGTA (0-μM Ca^2+^) or 5-mM HEDTA (1- and 10-μM Ca^2+^). No Ca^2+^ buffer was used for 100-μM Ca^2+^ solutions.

In symmetrical K^+^, macroscopic BK currents were elicited using a voltage protocol stepping from holding potentials of −100 or −150 to +350 mV (in +10 mV increments) for 20 ms and back to −80 mV for 10 ms to generate tail currents. Conductance–voltage (G–V) curves were obtained by measuring the instantaneous tail current amplitudes 200 μs after the start of the −80-mV step, dividing the current amplitude by the K^+^ driving force, and normalizing to the maximum conductance (G_max_) and plotting against the activating voltage step. Driving force was calculated from the voltage of the tail step (V_m_) subtracted by equilibrium potential for K^+^ (V_eq_). In symmetrical K^+^ conditions, V_eq_ for K^+^ is 0 mV, and V_eq_ is −80 mV for physiological K^+^ conditions. The half maximal voltage of activation (V_1/2_) was determined by fitting G–V curves to a Boltzmann function: $G={\left\{1+e^{(V_{1/2}-V)/k}\right\}}^{-1}$ in Origin 8.5 (OriginLab Corp., Northampton, MA, USA), where V is the command voltage and k is the slope factor defined by zF/RT. Time constants of activation (τ Activation) and deactivation (τ Deactivation) were analyzed in pClamp 10.3 (Molecular Devices, San Jose, CA, USA). Activation kinetics were determined by fitting the rising phase of the outward currents to single exponential functions. To evoke currents for measuring deactivation, patches were subjected to 20-ms voltage steps to +200 mV from a holding potential of −100 mV, followed by 10-ms voltage steps from −200 to −50 mV (in +10-mV increments) to obtain tail currents. Deactivation kinetics were determined by fitting tail currents with single exponential functions. Leak currents were subtracted using a P/5 protocol with a subsweep holding potential of −120 mV as previously described (Shelley et al., 2013).

For dephosphorylation experiments, calf intestinal alkaline phosphatase (Alk P, Cat. #M0290S, New England Biolabs Inc., Ipswich, MA, USA) was warmed to room temperature and diluted to 10 U/ml in the internal bath solution (with 1-μM Ca^2+^), and currents were recorded from inside out patches in control bath solution, or bath solution containing Alk P, 1 min after, patches were excised. For redox experiments, the reducing agent dithiothreitol (DTT, 1 mM, cat. #2325, Invitrogen, Waltham, MA, USA) and oxidizing agent hydrogen peroxide (H_2_O_2_, 0.3%, Cat. #H1009, Sigma-Aldrich, St. Louis, MO, USA) were diluted to working concentrations in the internal bath solution (with 10-μM Ca^2+^). Baseline currents were recorded 1 min after patches were excised. After recording baseline currents, vehicle control-, DTT-, or H_2_O_2_-containing solution was perfused into the bath, and posttreatment currents were recorded 10 min later. For Mg^2+^ experiments, currents were recorded in 1- or 10-μM Ca^2+^ internal bath solutions that contained either 0-, 1-, or 3-mM Mg^2+^. Reagents were stored at −20°C (Alk P, DTT) or 4°C (H_2_O_2_) as per the manufacturers’ instructions, and all reagents were freshly diluted in the internal solution on the same day experiments were performed.

For physiological K^+^ experiments, the external (pipette) solution contained (in mM): 134 NaCl, 6 KCl, 1 MgCl_2_, 10 glucose, and 10 HEPES with pH adjusted to 7.4 with NaOH. The internal (bath) solution contained: 110 K-aspartate, 10 NaCl, 30 KCl, 10 HEPES, 1 MgCl_2_, 5 HEDTA, and 10-μM free-Ca^2+^, with pH adjusted to 7.2 with KOH. To generate G–V relationships, currents were evoked from a holding potential of −100 mV, followed by 20-ms voltage steps from −150 to +150 mV (in +10-mV increments), followed by a 10-ms tail step to −150 mV. G–V analysis was performed on the tail currents, and all current kinetics were obtained as described previously.

Following square waveform current recordings, BK currents were recorded in response to AP waveforms. Three types of representative AP commands were used to evoke BK current: neuronal (mouse suprachiasmatic nucleus; Shelley et al., 2013), cardiac (mouse sinoatrial node; Lai et al., 2014), and smooth muscle (mouse urinary bladder; kindly provided by Dr. Tom Heppner, University of Vermont). AP command protocol stimulus files were generated using representative AP waveforms from each AP type plotted in Excel 2010 (Microsoft Corporation, Redmond, WA, USA). The peak current elicited by each AP voltage command was normalized to the peak steady state current evoked by square waveforms used to measure the G–V relationship for each patch. The peak steady-state current for each patch occurred between +90 and +120 mV.

### Statistics

BK current analysis was performed using pClamp 10.3 (Molecular Devices, San Jose, CA, USA). Graphs were generated in Prism 8.0 (GraphPad Software, San Diego, CA, USA), and statistical analysis was performed using Prism 8.0 or Origin 8.5 (OriginLab Corp., Northampton, MA, USA). One-way ANOVA with Bonferroni *post hoc* test was used to compare of V_1/2_ values and AP-evoked current amplitudes between constructs within each Ca^2+^ condition. Paired *t*-tests were used to compare the V_1/2_ before and after application of redox reagents, and unpaired *t*-tests were used to compare the ΔV_1/2_ due to redox reagents between WT and SNP-containing channels within each treatment condition. For BK current kinetics analysis, two-way repeated-measures ANOVAs with Bonferroni *post hoc* tests were used to determine significance for time constants of activation and deactivation between WT and SNP-containing constructs across voltages. Statistical significance was achieved if *P* < 0.05. Data in figures are presented as the mean ± SEM.

## Results

### Predicting Non-synonymous Single Nucleotide Polymorphisms That Regulate BK Current Properties

The *KCNMA1* coding sequence is comprised of 29 constitutive and eight alternative exons spanning 768 kilobases on human chromosome 10 [Zemen et al., 2015; gene ID: 3778, National Library of Medicine (US), NCBI (2002)]. More than 150 SNPs in the human *KCNMA1* gene encoding the BK channel α subunit (gene ID: 3778) were identified from publicly available National Center for Biotechnology Information dbSNP datasets (Sherry et al., 2001). The focus of this study was to identify non-synonymous human *KCNMA1* SNPs that would result in BK channel gating alterations. To this end, 99 non-synonymous SNPs located within the coding region were evaluated according to the change in amino acid properties and predicted alterations in protein function based on previous mutagenesis studies and computational models. We primarily focused on SNPs within the cytoplasmic “gating ring” of the BK channel, which accounts for ~80% of the protein sequence and contains the Ca^2+^-binding domains that regulate Ca^2+^-dependent gating (Magleby, 2003; Salkoff, 2006; Lee and Cui, 2010). Modulations of gating ring sequence *via* insertion of alternative exons, or mutations like D434G, have been shown to significantly affect BK currents (Latorre et al., 2017). Furthermore, residues within several regions of the gating ring structure, including Mg^2+^- and Ca^2+^-binding sites as well as interaction sites between the two RCK domains, have been reported in previous studies to affect BK currents when altered (Latorre et al., 2017), suggesting SNPs within these regions would also have the potential to alter channel properties.

In addition to functional studies, BK channel protein sequences containing SNP substitutions were analyzed in MutPred2, a model that predicts deleterious effects of amino acid substitutions using sequence homology and structural information from protein databases (Li et al., 2009; Pejaver et al., 2017). To evaluate a mutation as deleterious, MutPred2 assigns the mutation a general score from 0 (benign) to 1 (pathogenic). As a control to verify MutPred2 as a tool to identify residue substitutions that can alter BK current properties, we determined MutPred2 scores for several previously characterized BK channel mutations with known effects. Two “control” mutations had relatively significant MutPred2 scores when introduced into the hBK sequence (hBK_QEERL_, GenBank MG279688). The mutation R272Q produced a high MutPred2 score of 0.780. This mutation has been shown to enhance voltage-dependent gating and left-shifts the G–V relationship to more hyperpolarized potentials (Díaz et al., 1998). In addition, D959A, which is located within the RCK2 Ca^2+^ bowl, and exhibits a +80 mV shift in the voltage-dependence of activation compared with WT channels (Bao et al., 2004), was assigned a medium MutPred2 score (0.649). In contrast, D434G mutation in the RCK1 domain had a relatively low MutPred2 score (0.173), despite the fact this mutation is causative for epilepsy and significantly left-shifts the G–V due to enhanced Ca^2+^ sensing (Du et al., 2005). These controls suggest that MutPred2 has some predictive value with residue substitutions that alter BK channel properties through different mechanisms, but with limitations, potentially due to a lack of additional structural interaction information. Therefore, we concentrated on SNPs located in specific regions of interest within the BK channel structure that had been previously shown to regulate BK current properties in functional studies. From there, we selected higher priority SNPs based on a MutPred2 score ≥0.5.

Based on their proximity to residues previously identified to regulate BK channel properties when mutated, we started with nonconservative SNPs C495G, N599D, and R800W, which all produced high MutPred2 scores (Figure 1A, Table 1). The C495G SNP would have the potential to alter protein conformation by introducing a flexible glycine residue into a flexible linker region in RCK1 that is conserved among BK channels (Jiang et al., 2001; Zhang and Horrigan, 2005). Deletion of this RCK1 linker has been shown to left-shift the G–V relationship of BK currents (Zhang and Horrigan, 2005). The D434G mutation, which lies in the N-terminal portion of RCK1 domain, has been shown to alter the flexibility of RCK1 and affects allosteric gating (Yang et al., 2010), suggesting other substitutions, like C495G, within this region could potentially alter BK channel gating properties. Furthermore, cysteine oxidation has been shown to modulate BK current properties. In previous studies, the mutation of C495 to alanine was shown to partially inhibit the effects of the oxidizing agent H_2_O_2_, which normally produces a large right-shift of the G–V relationship for WT currents (DiChiara and Reinhart, 1997; Zhang and Horrigan, 2005; Zhang et al., 2006). Additionally, C495A currents exhibited right-shifted V_1/2_ values at low (≤1 μM) Ca^2+^ concentrations compared with WT channels (Zhang and Horrigan, 2005). Taken together, these studies raise the possibility that the C495G SNP could potentially alter redox modulation and channel activity. The N599D SNP introduces a negative charge near a region of the RCK1 domain involved in Ca^2+^ coordination. N599D is directly adjacent E600, a residue critical to the Ca^2+^-sensing site in RCK1 (Xia et al., 2002; Zhang et al., 2010). Zhang et al. (2010) showed that E600A reduced the magnitude of the Ca^2+^-dependent shift in the V_1/2_ (ΔV_1/2_ between 0 and 100 μM Ca^2+^ conditions) to −100 mV, compared with the −200 mV leftward shift observed for WT currents. The R800W SNP, located within RCK2, eliminates a positive charge and introduces a larger, hydrophobic residue. R800W is next to G798 and N801, two residues regulating the flexible interface that governs interactions between RCK1 and RCK2 (Figure 1B; Kim et al., 2006, 2008). In a previous study, the G798D mutation caused a large hyperpolarizing shift in the G–V relationship at multiple Ca^2+^ concentrations, and N801K caused a −45-mV hyperpolarizing G–V shift, indicating the importance of amino acid substitutions in this region of the channel (Kim et al., 2008).

Additionally, SNP A138V was prioritized for further study based on its association with autism (Table 1), despite its relatively low MutPred2 score. A138V introduces a hydrophobic residue and is located near residues that coordinate Mg^2+^ binding between the S0–S1 linker and RCK1 domain within the interface between the voltage sensor and RCK1 N-terminal lobe (Yang et al., 2008; Hite et al., 2017), suggesting A138V could alter BK channel function and Mg^2+^-dependent gating. In total, four novel SNP substitutions were introduced into the BK channel sequence to test their effects on current properties.

### Effect of Single Nucleotide Polymorphisms on BK Current Properties

**Figure 2:**
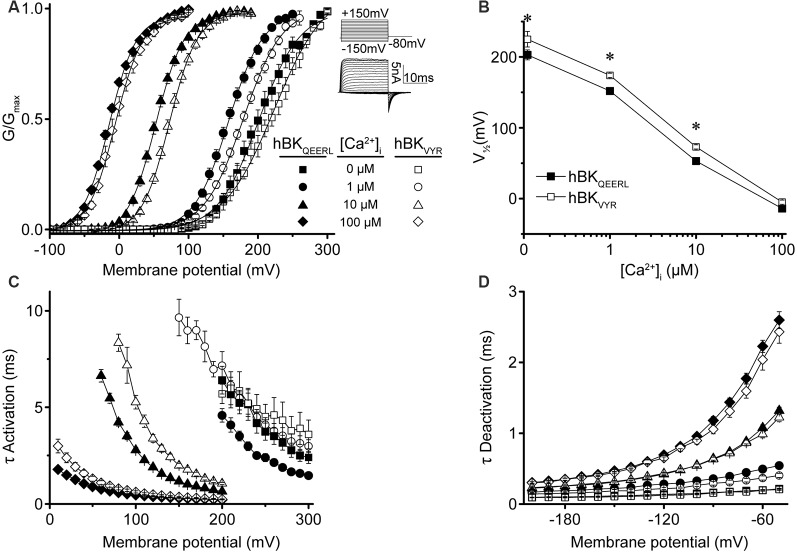
hBK_QEERL_ and hBK_VYR_ current properties in symmetrical K^+^. **(A)** G–V relationships for macroscopic hBK_QEERL_ and hBK_VYR_ currents recorded in symmetrical K^+^ at 0-, 1-, 10-, and 100-μM Ca^2+^. *Inset*: voltage-step protocol and representative currents from hBK_QEERL_ channels at 100-μM Ca^2+^. Currents were evoked from a holding potential of −100 mV using 20-ms voltage steps from −150 up to +300 mV, in +10 mV increments, followed by a 10-ms tail step to −80 mV. **(B)** V_1/2_ vs. Ca^2+^ relationship exemplifying the difference in voltage dependence of activation between hBK_QEERL_ and hBK_VYR_ at 0- (*P* = 0.006), 1- (*P* < 0.0001), and 10-μM Ca^2+^ (*P* = 0.002). *P < 0.05, two-way ANOVA with Bonferroni *post hoc* test between constructs across all Ca^2+^ conditions. **(C)** Time constants of activation (τ Activation) vs. voltage. hBK_VYR_ exhibited slower activation at 1- (*P* = 0.01), 10- (*P* < 0.0001), and 100-μM Ca^2+^ (*P* = 0.001). **(D)** Time constants of deactivation (τ Deactivation) vs. voltage at each Ca^2+^ concentration. Deactivation currents were elicited from a holding potential of −100 mV using 20-ms voltage steps to +200 mV, followed by 10-ms voltage steps from −200 to −50 mV (in +10 mV increments). hBK_VYR_ exhibited faster deactivation at 1-μM Ca^2+^ (*P* = 0.003). Differences in current kinetics were determined using two-way repeated measures ANOVAs with Bonferroni *post hoc* tests. *N* = 8–28 recordings per channel variant at each Ca^2+^ concentration.

We determined the effects of these SNPs on steady-state BK current properties in symmetrical K^+^ across four standard concentrations of intracellular (bath) Ca^2+^ from BK channel constructs transfected into HEK293T cells. Since BK channels acquire tissue-specific functional diversity through alternative splicing, two different human BK splice variant backgrounds were used, hBK_QEERL_ and hBK_VYR_ (Figure 2), designated based on their C-terminal alternate exon sequences (alternate splice site 4). The hBK_QEERL_ variant lacks insertions at the first three alternative splice sites in the gating ring and is expressed in multiple tissues, including human vascular smooth muscle and brain (Dworetzky et al., 1994; Pallanck and Ganetzky, 1994; McCobb et al., 1995). The hBK_VYR_ channel variant contains alternative exons at sites 1 and 3 and was previously cloned from human, chick, and turtle tissues (Pallanck and Ganetzky, 1994; Tseng-Crank et al., 1994; Rosenblatt et al., 1997; Jones et al., 1999). BK_VYR_ has been shown to undergo alternative-exon-dependent phospho-regulation (Shelley et al., 2013).

First, conductance–voltage (G–V) relationships produced by hBK_QEERL_ and hBK_VYR_ were compared with each other (Figure 2A). In symmetrical K^+^, hBK_QEERL_ currents exhibited G–Vs that were left-shifted to more hyperpolarized potentials at <100-μM Ca^2+^, compared with hBK_VYR_ currents (Figure 2A). The differences in the voltage of half maximal activation (V_1/2_) were most apparent in 1- and 10-μM Ca^2+^, where hBK_QEERL_ values were left-shifted by −27 mV (*P* < 0.0001) and −18 mV (*P* = 0.002), respectively (Figure 2B). In addition, hBK_QEERL_ currents had faster activation time constants (τ Activation), while deactivation time constants (τ Deactivation) were mostly unchanged (Figures 2C,D). Thus, these two human BK splice variants were distinguishable in their current properties.

**Figure 3:**
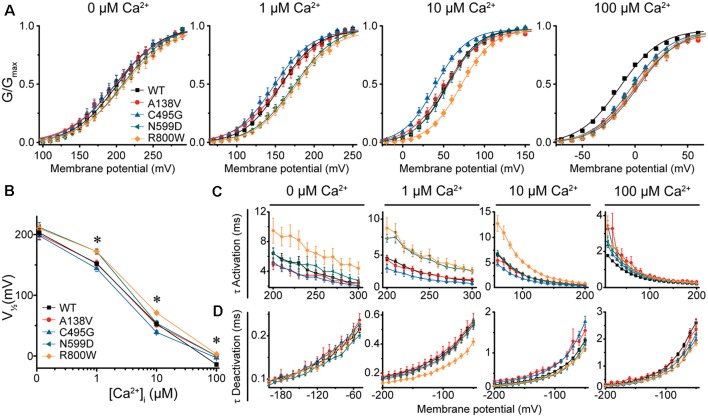
BK current properties from SNP-containing hBK_QEERL_ channels. **(A)** G–V relationships for BK currents recorded in symmetrical K^+^ at 0-, 1-, 10-, and 100-μM Ca^2+^ for control wild-type (WT) hBK_QEERL_ channels and channels containing A138V, C495G, N599D, or R800W SNP substitutions. Voltage protocols were identical to those in Figure 2. **(B)** V_1/2_ vs. Ca^2+^ plot demonstrating differences in voltage dependence of activation between WT and SNP-containing channels. Significant differences were found at 1- (N599D, *P* = 0.02; R800W, *P* = 0.03), 10- (C495G, *P* = 0.02; R800W, *P* < 0.0001), and 100-μM Ca^2+^ (A138V, *P* = 0.001; C495G, *P* = 0.009; N599D, *P* = 0.0001; R800W *P* < 0.0001). *P < 0.05, one-way ANOVA with Bonferroni *post hoc* test. **(C)** τ Activation vs. voltage at each Ca^2+^ concentration. Significant differences in activation kinetics between WT and SNP-containing channels were observed at 0- (R800W, *P* = 0.03), 1- (C495G, *P* = 0.03; N599D, *P* = 0.0001; R800W, *P* = 0.002), 10- (C495G, *P* = 0.002; R800W, *P* < 0.0001), and 100-μM Ca^2+^ (A138V, *P* = 0.0006; C495G, *P* = 0.0007; N599D, *P* = 0.001; R800W, *P* < 0.0001). **(D)** τ Deactivation vs. voltage at each Ca^2+^. Significant differences in deactivation kinetics were found between WT and SNP-containing channels at 1- (R800W, *P* = 0.001), 10- (A138V, *P* = 0.002; C495G, *P* = 0.0005), and 100-μM Ca^2+^ (C495G, *P* = 0.0001; N599D, *P* = 0.02; R800W, *P* < 0.0001). Significant differences (*P* < 0.05) in current kinetics between WT and each SNP-containing construct were determined using two-way repeated measures ANOVAs at each Ca^2+^. *N* = 5–28 recordings per construct at each Ca^2+^ concentration.

Next, SNP substitutions A138V, C495G, N599D, and R800W were initially introduced into the hBK_QEERL_ variant background. BK currents from each channel variant exhibited robust activation upon membrane depolarization or elevation of intracellular Ca^2+^ (Supplementary Figure S1). The largest effects due to introduction of the SNPs were observed at 1- and 10-μM Ca^2+^ (Figures 3A,B). R800W consistently exhibited loss-of-function properties based on the depolarizing shift in the V_1/2_ in combination with decreased activation and increased deactivation rates. At 1-μM Ca^2+^, R800W currents exhibited a depolarizing right shift (+20 mV) in the G–V relationship (*P* = 0.03), slower activation (*P* = 0.002), and faster deactivation (*P* = 0.001) compared with WT hBK_QEERL_ currents (Figures 3A–D). In keeping with this effect, at 10-μM Ca^2+^, R800W currents exhibited a significantly right-shifted G–V relationship (+18 mV, *P* = 0.03) and slower activation (*P* < 0.0001) compared with WT hBK_QEERL_. This suggests R800W hinders the activation of BK channels.

In contrast, C495G currents exhibited some properties characteristic of gain-of-function. Compared with WT hBK_QEERL_, C495G currents were significantly left-shifted to more hyperpolarized potentials at 10-μM Ca^2+^ (by −14 mV, *P* = 0.02), suggesting C495G facilitates the opening of BK channels. C495G currents exhibited slightly faster activation (*P* = 0.002) and slower deactivation (*P* = 0.0005) at 10-μM Ca^2+^ compared with WT hBK_QEERL_ currents (Figures 3A–D). Similarly, at 1-μM Ca^2+^, C495G currents exhibited a slight hyperpolarizing, albeit not statistically significant, shift in the G–V relationship (−8 mV, *P* > 0.05) along with faster activation (*P* = 0.03) compared with WT hBK_QEERL_ currents (Figures 3A–C).

Unlike C495G and R800W, A138V and N599D had less consistent effects on BK currents at 1- and 10-μM Ca^2+^ (Figures 3A–D). N599D currents were not different from WT hBK_QEERL_ at 10-μM Ca^2+^. However, at 1-μM Ca^2+^, N599D currents exhibited a rightward G–V shift (+20 mV, *P* = 0.02) compared with WT hBK_QEERL_ currents, as well as slower activation (*P* = 0.0001), suggesting that N599D does not facilitate channel opening at 1-μM Ca^2+^. The G–V relationship and current kinetics for A138V currents at 1- and 10-μM Ca^2+^ were not significantly different from WT hBK_QEERL_ currents.

At high Ca^2+^ concentrations (100 μM), the G–V relationships for A138V, C495G, N599D, and R800W currents were all significantly right-shifted to more depolarized potentials compared with WT hBK_QEERL_ currents (by +11 to +17 mV; Figures 3A,B). Consistent with the rightward G–V shifts, A138V, C495G, N599D, and R800W currents exhibited varying degrees of slower activation and faster deactivation at 100-μM Ca^2+^ (Figures 3C,D). Finally, at 0-μM Ca^2+^, there were no significant differences in the G–V relationships or current kinetics between WT hBK_QEERL_ and A138V, C495G, and N599D-containing channels (Figures 3A–D). Although the G–V relationship was not significantly different between WT hBK_QEERL_ and R800W, we found that R800W currents still exhibited slower activation kinetics (*P* = 0.03; Figure 3C).

Taken together, these results show that candidate SNPs could affect multiple aspects of BK current properties. We found that C495G and R800W had the most consistent effects on BK currents. As predicted by high MutPred2 scores, C495G, N599D, and R800W affected current activation and kinetics to varying degrees across Ca^2+^ conditions. Furthermore, both gain- and loss-of-function changes could be detected. Simplistically, but with a notable exception (at 100-μM Ca^2+^), C495G produced mostly increases in channel activity *via* left-shifts in the G–Vs at 1- and 10-μM Ca^2+^. Conversely, R800W could be summarized as decreasing channel activity *via* right-shifts of the G–Vs and slowed activation kinetics. N599D did not produce effects that could be themed as readily, with G–V shifts in both directions (1- and 100-μM Ca^2+^) or not at all (0- and 10-μM Ca^2+^). Unlike C495G, N599D, and R800W, SNP A138V produced the fewest changes in activation and kinetics across Ca^2+^ conditions, correlating with the lower MutPred2 score.

### Effect of Single Nucleotide Polymorphisms on Alternatively Spliced and Posttranslationally Modified BK Channels

**Figure 4:**
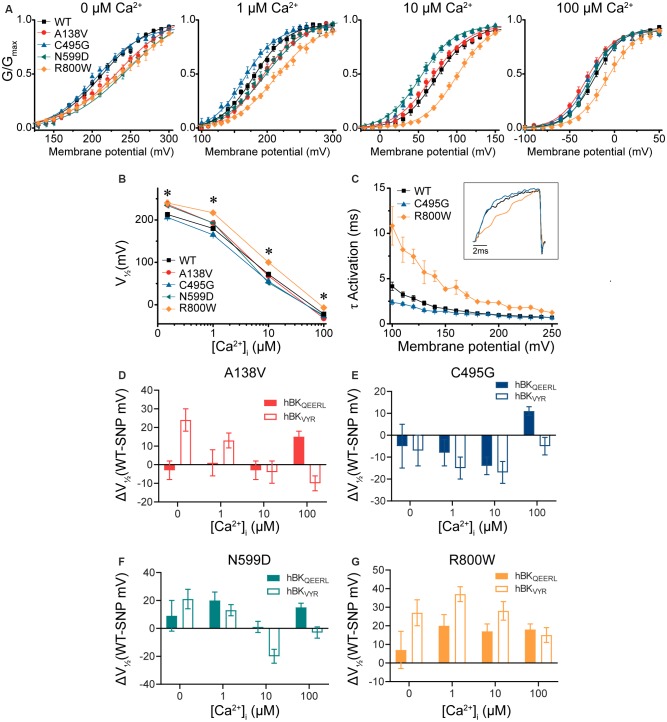
BK current properties from SNP-containing hBK_VYR_ channels. **(A)** G–V relationships from BK currents recorded in symmetrical K^+^ solutions at 0-, 1-, 10-, and 100-μM Ca^2+^ from control WT hBK_VYR_ channels and channels containing A138V, C495G, N599D, and R800W SNP substitutions. Voltage protocols identical to those in Figure 2. **(B)** V_1/2_ vs. Ca^2+^ relationship. Significant differences in V_1/2_ values between WT and SNP-containing channels were found at 0- (A138V, *P* = 0.006; N599D, *P* = 0.03; R800W, *P* = 0.001), 1- (A138V, *P* = 0.041; C495G, *P* = 0.03; N599D, *P* = 0.03; R800W, *P* < 0.0001), 10- (C495G, *P* = 0.02; N599D, *P* = 0.0008; R800W, *P* < 0.0001), and 100-μM Ca^2+^ (R800W, *P* = 0.003). *P < 0.05, one-way ANOVA with Bonferroni *post hoc* tests comparing constructs at each Ca^2+^ concentration. **(C)** τ Activation vs. voltage for WT, C495G, and R800W at 10-μM Ca^2+^. *Inset*: normalized current traces from each construct evoked by a 20-ms voltage step to +100 mV. Compared with WT, C495G exhibited faster activation kinetics (*P* = 0.002), while R800W exhibited slower activation kinetics (*P* < 0.0001). *P < 0.05, two-way repeated-measures ANOVA. **(D–G)** Summary of the difference in the average V_1/2_ values (ΔV_1/2_ in mV) between WT and SNP-containing channels for hBK_QEERL_ and hBK_VYR_ variant backgrounds. *N* = 8–21 recordings per construct at each Ca^2+^ concentration.

To determine the consistency of these SNP effects on BK channel function in the presence of alternative splicing, we introduced these four SNPs into another BK channel splice variant, hBK_VYR_ (Shelley et al., 2013). Using identical voltage protocols and symmetrical K^+^ solutions, we evaluated the net effects of these SNPs on G–V relationships (Figure 4A). While hBK_VYR_ G–V relationships are right-shifted compared with hBK_QEERL_ at the same Ca^2+^ concentrations (Figure 2B), the SNPs still produced G–V changes in both directions (Figures 4A,B). At all Ca^2+^ concentrations, R800W produced a rightward G–V shift in hBK_VYR_ currents, consistent with a loss-of-function phenotype, while C495G exhibited a leftward G–V shift at 1- and 10-μM Ca^2+^, indicating enhanced channel activation (Figure 4A). The shifts in G–V caused by either R800W and C495G, as denoted by the ΔV_1/2_, were generally consistent on both splice variant backgrounds (Figures 4E,G). The effect of these two SNPs on hBK_VYR_ activation kinetics due to the G–V shifts also corroborated the effects observed on the hBK_QEERL_ background, as C495G accelerated the activation kinetics at negative voltages and R800W consistently slowed activation kinetics (as exemplified at 10-μM Ca^2+^ in Figure 4C) compared with WT hBK_VYR_ at the equivalent voltages.

The effects of A138V and N599D were not consistent within individual Ca^2+^ conditions between the hBK_VYR_ and hBK_QEERL_ backgrounds (Figures 4D,F). On the hBK_VYR_ variant background, A138V and N599D exhibited a rightward G–V shift at 0- and 1-μM Ca^2+^, while N599D exhibited a leftward G–V shift at 10-μM Ca^2+^. While these data show that SNP effects are clearly discernable on two distinct splice variant backgrounds, the specific effects can be inconsistent. Therefore, we focused our continued evaluations on C495G and R800W, the two SNPs that produced the most consistent effects across Ca^2+^ conditions and splice variant backgrounds.

**Figure 5:**
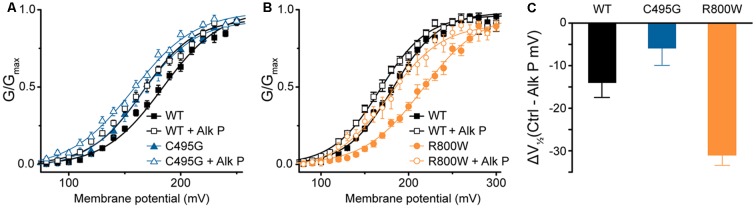
Effects of dephosphorylation on C495G and R800W hBK_VYR_ currents. **(A,B)** G–V relationships from BK currents recorded in symmetrical K^+^ at 1-μM Ca^2+^ for control hBK_VYR_ (WT) and C495G **(A)** or R800W **(B)** channels in control conditions or in the presence of dephosphorylating agent alkaline phosphatase (Alk P) in the intracellular solution. Voltage protocols were identical to those used in Figure 2. Alk P treatment was associated with a significant leftward G–V shift for each construct (WT, *P* = 0.03; C495G, *P* < 0.0001; R800W, *P* < 0.0001). C495G + Alk P was significantly different compared with WT + Alk P (*P* < 0.0001). R800W + Alk P was significantly different compared to WT + Alk P (*P* = 0.02). Significance (*P* < 0.05) was tested using one-way ANOVA with Bonferroni *post hoc* test comparing V_1/2_ values for each construct between control and Alk P conditions and between WT and SNPs constructs within each treatment condition. **(C)** ΔV_1/2_ plot summarizing the magnitude of the V_1/2_ shift due to Alk P for each construct (Average V_1/2_ control − Average V_1/2_ + Alk P). *N* = 9–20 recordings per construct for each treatment condition.

Besides alternative splicing, posttranslational modifications are known to modify BK channel properties (Kyle and Braun, 2014). One such modification is phosphorylation, and we previously showed that mouse BK_VYR_ currents exhibited left-shifted G–Vs when subjected to dephosphorylation by alkaline phosphatase (Shelley et al., 2013). In the current study, G–V relationships for human hBK_VYR_ currents were also left-shifted when alkaline phosphatase was applied to the intracellular side of the patches (Figures 5A–C), allowing a test of whether SNP effects persist under conditions where the difference in channel properties is produced by a posttranslational mechanism. The G–V for C495G currents in control conditions was similar to the G–V relationship of WT hBK_VYR_ currents recorded in the presence of alkaline phosphatase (Figure 5A). However, exposing C495G channels to alkaline phosphatase still produced a further left-shift of the G–V (*P* < 0.0001). Similarly, the effect of alkaline phosphatase was not precluded by R800W and still resulted in a left-shift of the G–V compared with baseline R800W currents (*P* < 0.0001; Figure 5B). However, the relative magnitude of the G–V shift (ΔV_1/2_) due to alkaline phosphatase was reduced for C495G and increased for R800W compared with WT hBK_VYR_ currents (Figure 5C). These results suggest that when these mechanisms act in the same direction, as with the left-shifting effect of dephosphorylation on the already left-shifted C495G current G–V, a relative maximum effect may be reached, potentially due to these mechanisms acting through the same pathway. On the other hand, R800W exerts at least some of its effects on BK channel properties independently of channel phosphorylation, since the G–V relationship for R800W in alkaline phosphatase is still right-shifted compared with WT. However, because the ΔV_1/2_ for R800W is actually larger than WT, it also suggests that this residue could potentially interact with a nearby phospho-regulated residue.

### Interaction of Multiple Single Nucleotide Polymorphisms Within a Single BK Channel Subunit

**Figure 6:**
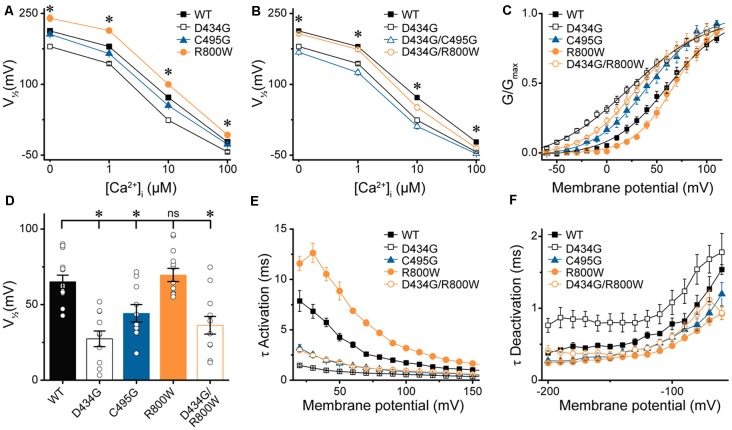
Effects of SNP substitutions in parallel with the D434G mutation on hBK_VYR_ currents. **(A,B)** V_1/2_ vs. Ca^2+^ relationships for currents evoked from control hBK_VYR_ (WT), D434G, C495G, and R800W channels **(A)** and channels containing SNPs in parallel with the D434G mutation in the same BK α subunit **(B)** recorded at 0-, 1-, 10-, and 100-μM Ca^2+^ in symmetrical K^+^ using the same voltage protocols as those in Figure 2. **(C)** G–V relationships for BK currents recorded in physiological K^+^ solutions at 10-μM Ca^2+^ for WT, D434G, C495G, R800W, and D434G/R800W channels using the voltage protocols in Supplementary Figures S2A,C. **(D)** V_1/2_ values for each construct obtained from the G–V relationships in **(C)**. **(E,F)** τ Activation **(E)** and τ Deactivation **(F)** for WT and SNP-containing channels in physiological K^+^. Significant differences in kinetics were observed between WT and SNP-containing channels for activation (D434G, *P* < 0.0001; C495G, *P* < 0.0001; R800W, *P* < 0.0001; D434G/R800W, *P* < 0.0001) and deactivation (D434G, *P* = 0.01; C495G, *P* = 0.02; R800W, *P* = 0.0006). *P < 0.05, one-way ANOVA with Bonferroni *post hoc* test for significant differences in V_1/2_ values between all constructs at each Ca^2+^ concentration **(A,B)**. Significant differences (*P* < 0.05) in current kinetics were tested with two-way repeated measures ANOVAs between WT and each SNP-containing construct across all voltages **(E,F)**. ns, not significant, *N* = 8–21 recordings per construct per Ca^2+^ concentration.

At present, one mutation in the BK channel coding sequence, D434G, has been linked to a human disease through familial pedigree, supporting the causative role of this mutation in epilepsy (Du et al., 2005). Because the seizure disorder shows variable penetrance (Li et al., 2018), we investigated whether further genetic variation could mitigate the functional effects of a known BK channel mutation. First, we determined whether the strong gain-of-function effects of the previously characterized D434G mutation were observable on the hBK_VYR_ variant background. In symmetrical K^+^, D434G-containing BK channels produced currents with G–V relationships that were significantly left-shifted compared with WT hBK_VYR_, R800W, and even C495G at every Ca^2+^ concentration (Figure 6A), corroborating the strong gain-of-function effect of the D434G mutation on a new splice variant background.

Next, we tested whether the C495G or R800W SNPs could influence the properties of BK channels containing the D434G mutation. C495G and R800W SNPs were each introduced in parallel with the D434G mutation within the same BK α subunit, and the resulting currents were first recorded in symmetrical K^+^ (Figure 6B). D434G/C495G currents were similar to the D434G mutation alone at 0-, 10-, and 100-μM Ca^2+^, suggesting the effect of combining these mutations is saturated. C495G is located near D434G, but the effect of D434G, which increases the allosteric coupling of gating ring movement by altering the flexibility of the AC region in RCK1 (Krishnamoorthy et al., 2005; Díez-Sampedro et al., 2006; Wang et al., 2009; Yang et al., 2010), could be dominant. However, at 1 μM Ca^2+^, the V_1/2_ of D434G/C495G currents became slightly more left-shifted compared with D434G alone (−18 mV, *P* = 0.008). This suggests that at an intermediate Ca^2+^ concentration, the effects of two alterations within the RCK1 domain that enhance channel activation can be additive, postulating C495G could have a small potential to exacerbate the gain-of-function properties of D434G under some conditions. Similarly, R800W altered D434G activation when introduced in parallel (Figure 6B). The V_1/2_ of D434G/R800W currents was significantly right-shifted compared with D434G alone at 0- (*P* = 0.0003), 1- (*P* < 0.0001), and 10-μM Ca^2+^ (*P* = 0.004). Interestingly, the V_1/2_ for D434G/R800W currents were no longer significantly different from WT hBK_VYR_ at 0-, 1-, and 100-μM Ca^2+^ conditions. Thus, the effects of R800W on current properties appear to be more additive, with the net effect of abrogating the increased activation produced by D434G. These data show that SNP variation, in principle, could affect the properties of a disease-linked mutation.

### Effect of Single Nucleotide Polymorphisms on Action Potential-Evoked BK Currents in Physiological K^+^

To determine whether SNP-induced alterations in G–V relationships and current kinetics were evident in physiological conditions, currents from WT and SNP-containing hBK_VYR_ channels were recorded in physiological K^+^ solutions containing physiological concentrations of Na^+^ (10-mM internal/134-mM external) and K^+^ (140-mM internal/6-mM external; Figures 6C–F; Supplementary Figure S2A). Although a plethora of studies have examined the effect of C-terminal mutations on BK current properties in symmetrical K^+^, few have examined the properties of BK currents evoked from channels expressed in heterologous cells using physiological K^+^. Since C495G and R800W had the largest effect on the G–V relationships and current kinetics in multiple Ca^2+^ concentrations in symmetrical K^+^, we hypothesized that C495G and R800W would also alter BK current properties in physiological solutions, as well as in parallel with the gain-of-function mutation D434G.

To test this hypothesis, macroscopic BK currents first were evoked by square depolarizing voltage steps in physiological K^+^ solutions with 10-μM intracellular Ca^2+^, a reasonable Ca^2+^ concentration that would be experienced by BK channels coupled to native Ca^2+^ sources in muscle and neurons (Fakler and Adelman, 2008). In the physiological K^+^ condition, D434G exhibited the same constellation of strong gain-of-function changes in BK current properties first revealed in symmetrical K^+^—a significantly left-shifted G–V (Figure 6C), with a ΔV_1/2_ of −38 mV (V_1/2_ D434G—V_1/2_ WT; *P* < 0.0001; Figures 6C,D), faster activation (*P* < 0.0001; Figure 6E), and slower deactivation (*P* = 0.01; Figure 6F) compared with WT. C495G left-shifted the G–V relationship and reduced the V_1/2_ (*P* = 0.048; Figures 6C,D), while R800W did not exhibit the same right-shift in the G–V relationship at all voltages observed in symmetrical K^+^ (Figures 6C,D). However, C495G currents still exhibited faster activation (*P* < 0.0001), while R800W currents exhibited slower activation (*P* < 0.0001) and faster deactivation (*P* = 0.0006) compared with WT currents (Figure 6E, Supplementary Figures S2B,C), demonstrating the effects of these two SNPs on kinetics followed the trends observed in symmetrical K^+^ solutions (Figures 3, 4). Deactivation kinetics between WT, C495G, and D434G/R800W were not significantly different (Figure 6F). Under these conditions, the presence of R800W alongside D434G did not significantly shift the G–V or V_1/2_ of D434G/R800W channels compared with D434G alone (Figures 6C,D); however, activation was slowed (*P* < 0.0001), and deactivation was accelerated (*P* = 0.0006; Figures 6E,F). These data suggest that the ability of R800W to reduce activation and increase deactivation rates is not solely due to shifts in the G–V relationship but is due to changes in gating kinetics of the channel itself.

**Figure 7:**
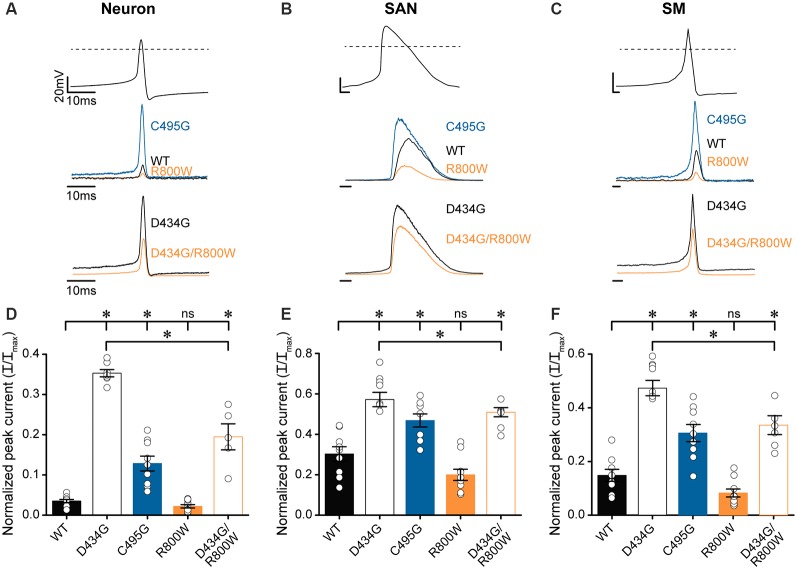
Effects of SNP substitutions on BK currents evoked by physiological stimuli. Action potential (AP)-evoked currents were recorded from WT and SNP-containing hBK_VYR_ channels in physiological K^+^ at 10-μM Ca^2+^. **(A–C)** Waveforms for Neuron **(A)**, SAN **(B)**, and SM **(C)** AP voltage command protocols (top traces) used to elicit BK currents from WT, D434G, C495G, R800W, and D434G/R800W channels (bottom traces). Current amplitudes are normalized to the maximum outward current evoked by the voltage step protocol in Supplementary Figure S2A. **(D–F)** Normalized peak AP-evoked BK current amplitudes from WT and SNP-containing channel variants activated by Neuron **(D)**, SAN **(E)**, and SM **(F)** voltage commands. Currents were significantly larger than WT for Neuron (D434G, *P* < 0.0001; C495G, *P* < 0.0001; D434G/R800W, *P* < 0.0001), SAN (D434G, *P* < 0.0001; C495G, *P* = 0.002; D434G/R800W, *P* = 0.0002), and SM (D434G, *P* < 0.0001; C495G, *P* = 0.0002; D434G/R800W, *P* < 0.0001) waveforms. D434G/R800W currents were reduced compared with D434G for Neuron (*P* < 0.0001), SAN (*P* = 0.02), and SM (*P* < 0.0001) waveforms. *P < 0.05, one-way ANOVA with Bonferroni *post hoc* test for significant differences between all current amplitudes for each AP command. ns, not significant; *N* = 6–11 recordings per construct.

After determining that D434G, C495G, and R800W regulate some or all biophysical properties of BK current activation in physiological K^+^, we tested the hypothesis that SNPs may alter BK currents evoked by native voltage stimuli, such as an AP (Figure 7). To test this hypothesis, the amplitudes of BK currents evoked by several different AP waveforms were measured (Figures 7A–C). The AP voltage commands were previously recorded from cells where BK channels regulate excitability: suprachiasmatic nucleus neurons of the hypothalamus, sinoatrial node cardiomyocytes, and bladder smooth muscle cells (Heppner et al., 1997; Montgomery and Meredith, 2012; Lai et al., 2014). These AP commands vary in the peak voltages and AP durations, providing a range of conditions to test the SNP effects.

AP-evoked D434G currents were significantly larger, with current magnitudes 2–10 times larger than WT currents, using all three physiological waveforms (Figures 7D–F). C495G also produced larger currents compared with WT from each AP command but not as large as D434G (Figures 7D–F). R800W produced a measurable reduction in current (by 34–45%), although not statistically significant, with each of the AP commands compared with WT. However, R800W expressed in parallel with the D434G mutation (D434G/R800W) significantly reduced the current compared with D434G alone (Figures 7D–F), indicating that the loss-of-function effect of R800W is more apparent in the presence of the increased currents produced by D434G. Taken together, these data reveal the consequences for hyperactive BK channel activity caused by the D434G mutation and the potential for SNPs such as C495G and R800W to affect BK current in a physiological context.

### Mechanistic Investigation of Single Nucleotide Polymorphism Effects on BK Channel Properties

**Figure 8:**
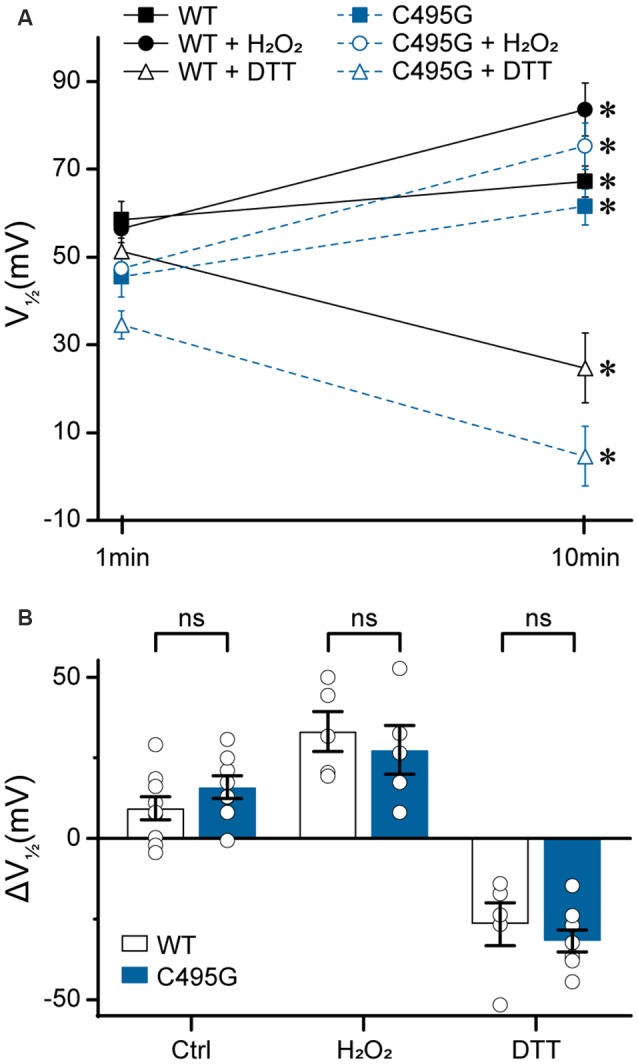
Effects of C495G on redox modulation of BK currents. Experiments were performed on the hBK_VYR_ splice variant background in symmetrical K^+^ solutions at 10-μM Ca^2+^. Voltage protocols were identical to those in Figure 2. **(A)** V_1/2_ values obtained from BK currents at 1 min (baseline) and 10 min after patch excision in vehicle control (Ctrl), H_2_O_2_, and DTT treatment conditions. After 10 min in Ctrl, WT (*P* = 0.03) and C495G (*P* = 0.003) channels both exhibited an increase in the V_1/2_ compared with baseline. At 10 min after application of oxidizing agent H_2_O_2_ (0.3%), WT (*P* = 0.006) and C495G (*P* = 0.007) exhibited a significant increase in the V_1/2_ compared with baseline. Ten minutes after application of reducing agent DTT (1 mM), both WT (*P* = 0.02) and C495G (*P* = 0.0009) exhibited a significant decrease in the V_1/2_ compared with baselines. *P < 0.05, paired *t*-tests for significant differences between baseline and posttreatment conditions for each construct. **(B)** Summary of the change in V_1/2_ (ΔV_1/2_ = V_1/2_ baseline—V_1/2_ 10 min) after 10 min in each condition. The ΔV_1/2_ of WT vs. C495G-containing channels was not significantly different in Ctrl, H_2_O_2_, or DTT conditions (*P* > 0.05). Unpaired *t*-tests were used for significant differences in ΔV_1/2_ values for WT and C495G within each treatment condition. ns, not significant; *N* = 5–9 recordings per construct per treatment condition.

**Figure 9:**
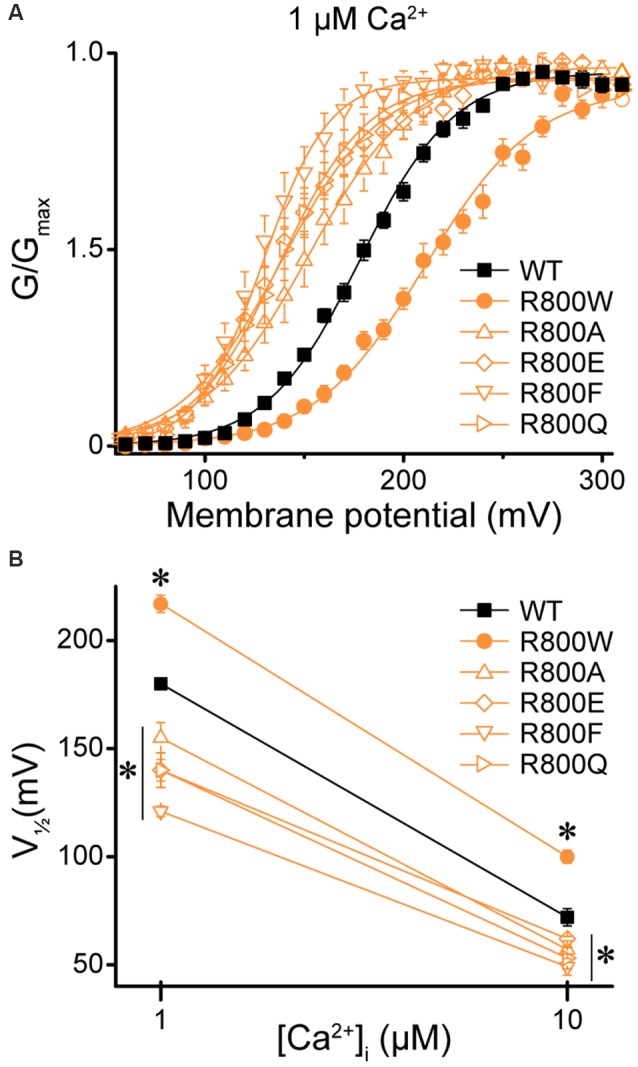
Effect of residue 800 size and charge on regulation of BK current activation. Experiments were performed on the hBK_VYR_ splice variant background in symmetrical K^+^ solutions. **(A)** G–V relationships between WT and channels containing R800 substitutions (R800A, R800E, R800F, R800Q, and R800W) at 1-μM Ca^2+^. Voltage protocols were identical to those in Figure 2. **(B)** V_1/2_ vs. Ca^2+^ plot for WT and SNP-containing channels at 1- and 10-μM Ca^2+^. Compared with WT, R800W exhibited a right G–V shift at 1- (*P* < 0.0001) and 10-μM Ca^2+^ (*P* < 0.0001), while all other R800 substitutions exhibited a left G–V shift at 1- (R800A, *P* = 0.002; R800E, *P* < 0.0001; R800F, *P* < 0.0001; R800Q, *P* < 0.0001) and 10-μM Ca^2+^ (R800A, *P* = 0.02; R800E, *P* = 0.06; R800F, *P* = 0.0002; R800Q, *P* = 0.007). *P < 0.05, one-way ANOVA with Bonferroni *post hoc* test comparing V_1/2_s between WT and all R800 substitution constructs at each Ca^2+^ condition. *N* = 5–21 recordings per construct per Ca^2+^ concentration.

**Figure 10:**
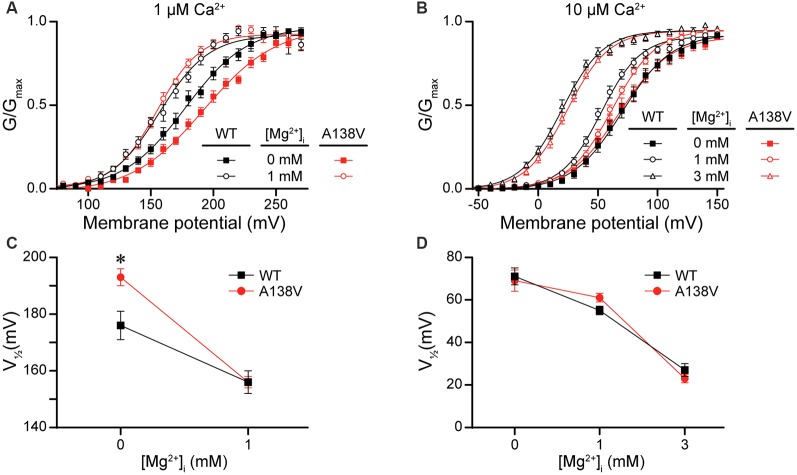
Effects of A138V on Mg^2+^-dependent activation of BK currents. Experiments were performed on the hBK_VYR_ splice variant background in symmetrical K^+^ solutions using the voltage protocols from Figure 2. **(A,B)** G–V relationships from WT and A138V channels in the presence of 0-, 1-, or 3-mM intracellular Mg^2+^ at 1- **(A)** or 10-μM Ca^2+^ **(B)**. **(C,D)** V_1/2_ vs. Mg^2+^ concentration plot for currents obtained in 1- **(C)** and 10-μM Ca^2+^ **(D)**. At 1-μM Ca^2+^, the V_1/2_ of WT and A138V currents were left-shifted in 1-mM Mg^2+^, compared with 0-mM Mg^2+^. At 10-μM Ca^2+^, the V_1/2_ values were left-shifted in 1- (WT, *P* = 0.003) and 3-mM Mg^2+^ (WT, *P* < 0.0001; A138V, *P* < 0.0001), compared with 0-mM Mg^2+^, and differences in V_1/2_ values between WT and A138V were eliminated. *P < 0.05, one-way ANOVA with Bonferroni *post hoc* comparing V_1/2_ values between the constructs at each Mg^2+^ condition and V_1/2_ values between Mg^2+^ conditions for each construct, within each Ca^2+^ concentration. *N* = 6–12 recordings per construct per treatment condition.

After establishing the range of effects BK current properties, additional experiments were designed to probe the potential mechanisms by which A138V, C495G, and R800W exert their effects on BK channel function (Figures 8–10). These studies were carried out using the hBK_VYR_ variant background. Previous studies had shown that cysteine oxidation at C495 in the BK channel inhibits BK currents (DiChiara and Reinhart, 1997; Zhang and Horrigan, 2005; Zhang et al., 2006), raising the possibility that elimination of C495 due to the SNP substitution C495G would protect the channel against the inhibitory effects of oxidation. It also suggests a mechanism for the enhanced activation exhibited by C495G channels. We tested this hypothesis by measuring the effects of oxidizing and reducing agents on the G–V relationship of WT and C495G currents.

First, we tested whether there was a time-dependent increase in the V_1/2_ following patch excision, which has been previously shown to occur due to BK channel oxidation that occurs over time in excised patches in the presence of reactive oxygen species (DiChiara and Reinhart, 1997). At 10 min following patch excision in control solutions, WT and C495G channels both exhibited an increase in V_1/2_ (WT, +9 ± 4 mV, *P* = 0.03; C495G +16 ± 4 mV, *P* = 0.003) compared with 1-min baselines, indicating both WT and C495G currents undergo a right G–V shift over time under these conditions (Figures 8A,B). Next, we tested whether application of oxidizing reagent H_2_O_2_ could further increase the V_1/2_ and whether application of reducing agent DTT could decrease the V_1/2_ of WT and C495G channels. If the C495G substitution reduces the ability of redox reagents to modify channel properties, then we would expect to see a decreased response of C495G currents to H_2_O_2_ and DTT compared with WT channels. Consistent with previous studies, we found H_2_O_2_ produced an increase in the V_1/2_ (+33 ± 6 mV, *P* = 0.006) for WT currents, while DTT produced a decrease (−22 ± 2 mV, *P* = 0.02; Figures 8A,B). Surprisingly, C495G also responded to these reagents with V_1/2_ shifts that were similar to WT, showing an increased V_1/2_ in the presence of H_2_O_2_ (+27 ± 6 mV, *P* = 0.007) and a decreased V_1/2_ (−30 ± 4, *P* = 0.0009) in DTT (Figure 8B). These data suggest that the gain-of-function effect conferred by C495G does not result solely from a reduction in oxidation of the BK channel.

Next, we investigated whether the loss-of-function effects of R800W, which substitutes an arginine for a tryptophan residue, could be recapitulated by other mutations. Previous studies have shown that changing the size, charge, and hydrophobicity of the amino acid residues that are located in the same region as the residue R800 can have a significant impact on BK channel gating properties due to chemical interactions forming a flexible RCK1–RCK2 interface (Kim et al., 2008). Four mutations were evaluated to determine if they could produce the large rightward shift in the G–V relationship observed for R800W (Figure 9A). Introduction of either alanine (R800A), which eliminates charge and is a smaller residue, or glutamine (R800Q), which eliminates the charge but maintains residue size, induced a leftward G–V shift opposite to the effect of R800W (Figure 9A). Replacing the tryptophan with a phenylalanine (R800F), another bulky aromatic residue, surprisingly also produced a left-shifted G–V (Figure 9A). These shifts were observed at both 1- and 10-μM Ca^2+^ (Figure 9B). Therefore, the loss-of-function effects of the R800W SNP are not simplistically dependent upon the change in the size, charge, and hydrophobicity of this substituted residue, and it is possible that the tryptophan substitution forms a unique tertiary interaction.

Lastly, we hypothesized that the A138V SNP, which exhibited variable but detectable effects on current properties (Figures 3, 4D), might influence Mg^2+^-dependent gating of the BK channel, due to the proximity of A138 to the Mg^2+^ coordination residue (D164; Yang et al., 2008). To test this hypothesis, we examined BK current activation from WT and A138V channels in the presence of 0-, 1-, and 3-mM intracellular Mg^2+^ at two different concentrations of intracellular Ca^2+^. First, in the absence of Mg^2+^, A138V currents exhibited a right-shifted G–V and a more depolarized V_1/2_ (by +17 mV, *P* = 0.02) compared with WT currents at 1-μM Ca^2+^ (Figures 10A,C). Addition of 1-mM Mg^2+^ shifted both WT and A138V G–V relationships to more hyperpolarized potentials at 1-μM Ca^2+^ (WT, Mg^2+^-dependent ΔV_1/*2*_ = −20 mV, *P* = 0.002; A138V, ΔV_1/2_ = −37 mV, *P* < 0.0001). This leftward shift in the V_1/2_ value in the presence of 1-mM Mg^2+^ was larger for A138V currents than for WT currents, which eliminated the net difference between WT and A138V in 1-μM Ca^2+^/1-mM Mg^2+^ conditions (Figures 10A,C). However, at 10-μM Ca^2+^, no differences were observed between WT and A138V currents in the absence or presence of Mg^2+^, and the Mg^2+^-dependent shifts in the V_1/2_ values were similar at 1- and 3-mM Mg^2+^(Figures 10B,D). Although these data suggest A138V that could potentially alter the sensitivity of BK currents to Mg^2+^-dependent activation depending on the Ca^2+^ concentration, A138V does not eliminate Mg^2+^-dependent gating of the BK channel.

## Discussion

SNP variation has been implicated in a wide variety of human traits and disease risks, predicting that SNPs would alter protein function in measurable ways. However, the data linking specific SNPs to human phenotypes is incomplete for most gene sequences. Moreover, a major limitation to using allele frequency as a parameter to select SNPs for functional studies is limited or absent allele frequency information in the common databases. For example, the vast majority of non-synonymous SNPs reported for KCNMA1 to date are classified as “rare” (Richards et al., 2015), represented by only a single variant present in the database (Supplementary Table S1). As such, to assess whether *KCNMA1* SNPs might alter BK channel function in the absence of comprehensive genotype–phenotype data, we examined the 99 non-synonymous SNP variations in the BK channel for those predicted to alter channel properties based on the nature of the substitution, prior mutagenesis studies, and in one case, potential disease linkage. Focusing on four candidate SNPs, we investigated their effects on BK currents under a broad array of voltage, Ca^2+^, BK channel sequence variation (alternative splice variants and epilepsy mutation), and posttranslational conditions. These data first show that a predictive approach was able to identify some functionally consequential SNP residues in the human BK channel, which would not have been possible by evaluating the limited allele frequency data currently available for these residues (Supplementary Table S1). Our results further illustrate how these four SNP sequence variations modulate BK current properties, adding this type of genetic regulatory mechanism to the array of factors potentially influencing BK channel activity *in vivo*.

Although the mechanistic interpretations from this study are limited, the location of the SNPs and their functional consequences reveal some potentially new residues influencing gating. The C-terminus “gating ring” contains two RCK domains, which harbor the divalent cation-binding sites that modulate channel gating (Cui et al., 2009). RCK2 contains the high-affinity “Ca^2+^ bowl”, and RCK1 contains a second Ca^2+^-binding site and two residues that contribute to the Mg^2+^-binding site (Xia et al., 2002; Yang et al., 2007, 2008). Mg^2+^ is also bound by two residues located in the S0–S1, proximal to A138V, and S2–S3 intracellular loops (Yang et al., 2008). Because A138V altered the magnitude of the Mg^2+^-dependent shift in the current–voltage relationship under at least one condition, it suggests this residue could interact with the Mg^2+^-dependent allosteric gating mechanism. For the C495G SNP, we conclude that this SNP may act *via* altering the interactions between nearby residues, not *via* a redox mechanism, as previous mutagenesis studies would have predicted (Tang et al., 2004; Zhang and Horrigan, 2005; Zhang et al., 2006). C495 is within a series of eight amino acids in RCK1 that links an α helix and β sheet (Supplementary Figure S3), and this αD–βD linker is conserved among BK channels, but not present in other K^+^ channels (Zhang and Horrigan, 2005). Because deletion of the linker causes a left-shift in the G–V relationship, similar to the C495G SNP substitution, it suggests that modifications of C495 could disrupt the linker, or the linker’s interactions, with other residues in the gating ring (Zhang and Horrigan, 2005). The glycine residue could potentially make the αD–βD linker more flexible and alter the structure of the gating ring in the presence of Ca^2+^. Further studies for C495 mutations to residues that convey less flexibility could test this hypothesis.

The effects of mutating N599 to alanine have been previously reported in the mouse BK channel (N534A in a previous study), which had a relatively small effect on the G–V relationship compared with mutations at E600 (mouse E535A), a neighboring residue critical for Ca^2+^ sensing in RCK1 (Zhang et al., 2010). The negative charge introduced by N599D could disrupt the interaction of the RCK1 Ca^2+^-sensing site with other residues within the gating ring and, therefore, alter the G–V relationship and activation kinetics. However, N599D also affected current properties in the absence of Ca^2+^; therefore, further studies would be required to understand the structural mechanism underlying N599D alterations in BK current properties.

R800W is located near a proposed flexible interface between RCK1 and RCK2 (Figure 1B). Mutations in the vicinity of R800W in the rat BK channel (G803D and N806K) cause a left shift in the G–V relationship and an increase in the single channel open probability (Kim et al., 2008). With the R800W SNP, elimination of the positive charge and addition of a large, bulky tryptophan residue could potentially disrupt the RCK1/RCK2 flexible interface, therefore affecting structural changes in the gating ring. We found that the tryptophan residue contributes distinctive properties to cause a right-shift of BK currents, whereas other substitutions produced the opposite effect on the G–V relationship, suggesting complex interactions involving R800.

A caveat to this study is that the BK channel constructs contain a Myc tag at the N-terminus and a YFP tag inserted at the beginning of the RCK2 domain. Deletion of these tags from WT channels reveals no difference in the conductance–voltage relationships in the presence or absence of Ca^2+^ (Supplementary Figure S4), suggesting they do not alter BK channel function. However, some studies have shown that introduction of large fluorescent protein tags, depending on the site, can produce changes in BK current properties or prevent functional channel expression (Meyer and Fromherz, 1999; Giraldez et al., 2005). Larger tags at the N-terminus, such as GFP, could affect the currents (Meyer and Fromherz, 1999), but this protein insertion is much larger than the Myc tag used in this study. Previous studies incorporating small tags into the N-terminus either did not show such differences or the differences were small and not statistically significant (Wallner et al., 1996; Pratt et al., 2017). In another study, insertion of fluorescent protein tags into the RCK1–2 linker at three sites similar to the YFP insertion site in this study produced nonsignificant V_1/2_ differences that were predominantly less than 11 mV compared with untagged channels, with the maximum difference being 18 mV in only one case (Giraldez et al., 2005). By comparison, the V_1/2_ differences between WT and SNP-containing channels that were significant in this study were larger (11–37 mV), suggesting introduction of SNPs produces the changes in BK current properties beyond what might be expected for YFP insertion alone. In addition, the effect of the D434G mutation has been assessed in studies employing both tagged and untagged channels. The data in our study with this mutation are consistent in direction and magnitude with previous studies using untagged BK channels, including the leftward G–V shifts of 17–48 mV (Du et al., 2005; Lee and Cui, 2009; Wang et al., 2009), increased activation rates (~1.5- to 2-fold; Du et al., 2005; Lee and Cui, 2009; Wang et al., 2009), and decreased deactivation rates (~1- to 2-fold; Lee and Cui, 2009) between 0- and 100-μM Ca^2+^. In other studies, activation by voltage (Giraldez et al., 2005), Ca^2+^ (Giraldez et al., 2005; Miranda et al., 2013), and Mg^2+^ (Miranda et al., 2016) was similar for tagged vs. untagged BK channels. Although these data suggest that the YFP tag has little effect that would alter interpretation of this data from SNP-containing channels, it remains possible that they could potentially interfere with BK channel gating in ways not fully anticipated. Further studies using untagged BK channels would be necessary to elucidate the biophysical mechanisms through which these SNPs are exerting their effects on BK channel properties.

Lastly, our data provide an example for the functional consequences of introducing a SNP alongside a mutation within the same BK channel subunit. D434G is thought to increase channel activity by modulating the AC region, the N-terminal portion of the RCK1 domain which allosterically couples conformational changes in the cytosolic domain with the activation gate (Cui et al., 2009). D434G/C495G and D434G/R800W double mutants provide insight into whether the mechanism of action of C495G and R800W, which are located in the cytosolic gating ring, can produce additive effects with alterations in the AC region (D434G). The inability of D434G to completely preclude the G–V effects caused by C495G and R800W hints that, even though the flexibility of the AC region is reduced by D434G, the mutated domain can still translate the allosteric coupling between other distinct gating ring alterations and channel opening through another mechanism.

This study reveals that the details for SNP effects on BK current properties are complex and context dependent. Compared with a *bona fide* disease-associated mutation like D434G, SNP effects on BK currents were smaller and varied in both the magnitude and, sometimes, the direction. For example, the effects observed across two splice variant backgrounds in four Ca^2+^ conditions are summarized in Figures 4D–G. In contrast to the SNPs, the D434G mutation shows large gain-of-function effects in all conditions tested so far, corroborated by data presented in this study and several previous studies (Du et al., 2005; Wang et al., 2009; Yang et al., 2010). Such heterogeneity may promote the tolerance for sequence alterations associated with SNP variation, which may not be directly disease causing, as opposed to the more deleterious consequence of the D434G mutation, which causes seizure disorder (Du et al., 2005).

SNPs A138V and N599D showed the most variable effects across conditions, correlated with lower MutPred scores (Table 1) and locations in regions of more limited evolutionary conservation (Supplementary Figure S3). However, some of the alterations in BK current properties induced by A138V, such as the rightward-shift in the G–V relationships at 0–1-μM Ca^2+^, are consistent with the reduced BK current observed in cells obtained from an autistic patient (Laumonnier et al., 2006). Next, the evidence for C495G was somewhat more consistent. C495G had a higher MutPred score than A138V and N599D and also localized to a region of higher cross-species conservation. Most of the changes in BK current properties suggest that C495G has gain-of-function effects, culminating in a shift in the voltage-dependence of activation to more hyperpolarized potentials, speeding activation, and increasing AP-evoked currents in physiological K^+^. However, there was an exception to this theme at 100-μM Ca^2+^ that was specific to the hBK_QEERL_ background, for reasons that are not clear from the data presented here. This context dependence for C495G, as well as A138V and N599D, will need to be investigated with additional experiments designed to address the specific mechanism(s) by which each SNP residue alters gating.

In contrast to these SNPs, R800W consistently conferred varying degrees of loss-of-function properties on BK currents, shifting the voltage dependence of activation to more depolarized potentials and slowing activation. R800W has a high MutPred score and is highly conserved, even in invertebrate sequences (Supplementary Figure S3). Interestingly, despite corroboration of the loss-of-function characteristics in physiological K^+^ with standard voltage protocols, R800W did not significantly reduce the AP-evoked current compared with WT currents. A potential explanation for this result is that the experiments were performed on the hBK_VYR_ variant (Figure 6), which already exhibits a relatively right-shifted voltage dependence of activation compared with other splice variant backgrounds. The same AP commands elicited significantly reduced current amplitudes from R800W on the hBK_QEERL_ channel background compared with WT (normalized neuron AP-evoked current: WT, 0.32 ± 0.004; R800W, 0.015 ± 0.002; *P* = 0.002, *t*-test. SAN: WT, 0.26 ± 0.02; R800W, 0.18 ± 0.02; *P* = 0.0012, *t-test*) suggesting the loss-of-function effects of R800W become more apparent on a left-shifted channel background. Consistent with this, R800W significantly reduced the increased AP-evoked currents caused by the left-shifted D434G mutation, even in the context of the hBK_VYR_ variant (Figure 7).

Since BK channels are activated by a wide range of physiological stimuli between cell types, it could be hypothesized that the effects of BK channel SNPs on cellular excitability may be tissue specific. For example, the magnitude of the differences between WT and SNP-containing channels was more apparent using the neuronal AP command, which is shorter in duration, compared with the longer SAN AP command (Figure 7), providing an example of how SNP-induced changes to current kinetics may differentially influence channel function across tissues. The addition of auxiliary β and γ subunits, which can drastically shift the voltage dependence of BK currents, even in the absence of Ca^2+^, could further illuminate the impact of SNPs across different tissues (Li and Yan, 2016). The context-dependent differences in these SNP effects may be an important mechanism contributing to BK current diversity within the body or even between individuals within a population.

## Data Availability Statement

Datasets used in this study can be found in the Database of Single Nucleotide Polymorphisms (dbSNP). Bethesda (MD): National Center for Biotechnology Information, National Library of Medicine. dbSNP accession: rs144215383, rs201243440, rs140520584, rs199681253 and rs137853333. Available at: https://www.ncbi.nlm.nih.gov/snp.

## Author Contributions

ML, AP and AM designed the experiments and wrote the manuscript. ML and AP performed the experiments. ML, AP, JL and AM analyzed the data.

## Conflict of Interest

The authors declare that the research was conducted in the absence of any commercial or financial relationships that could be construed as a potential conflict of interest.

## Footnotes

^1^http://www.ncbi.nlm.nih.gov/projects/SNP/

^2^http://mutpred.mutdb.org/

^3^https://somapp.ucdmc.ucdavis.edu/pharmacology/bers/maxchelator/webmaxc/webmaxcS.htm
