# Supplementary material for: Effects of Single Nucleotide Polymorphisms in Human KCNMA1 on BK Current Properties
Source: Front Mol Neurosci. 2019 Dec 3;12:285. doi: 10.3389/fnmol.2019.00285 (PMC6901604; doi:10.3389/fnmol.2019.00285)
Supplement: Supplementary file 1 [file Data_Sheet_1.PDF]

Supplementary Material

Effects of single nucleotide polymorphisms in human *KCNMA1* on BK current properties

Amber E. Plante, Michael H. Lai, Jessica Lu, and Andrea L. Meredith

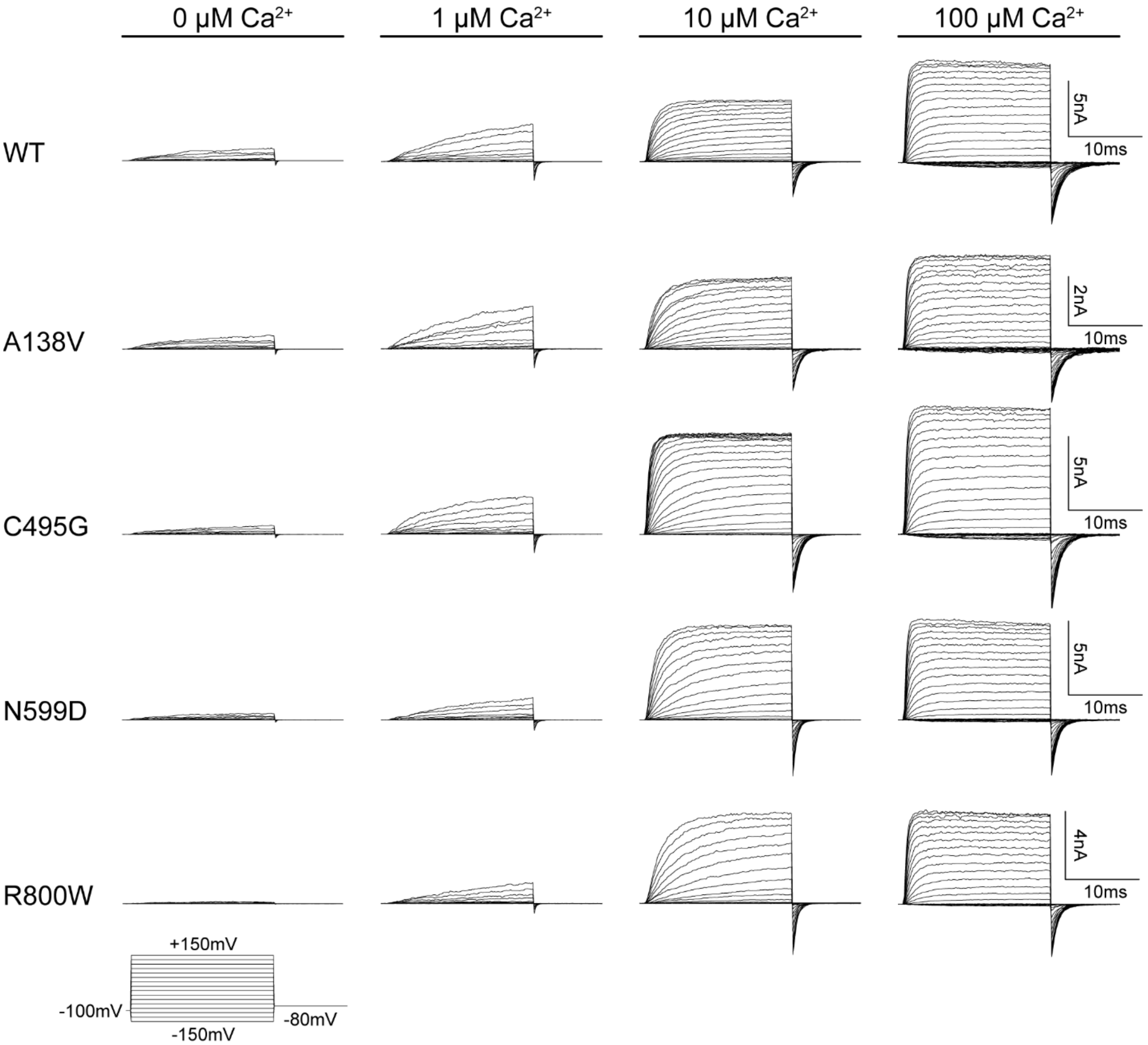

Figure S1. BK currents from WT and SNP-containing hBK channels in symmetrical K^+. Recordings were performed on the hBK_{QEERL} variant background. Representative macroscopic BK current traces from inside-out patches at 0, 1, 10 and 100 µM Ca^{2+} from control wildtype (WT), A138V, C495G, N599D, and R800W channels. Currents were evoked using a voltage protocol stepping from -150 to +300 mV in +10 mV increments (20 ms) from a holding potential of -100 mV, followed by a tail step to -80 mV (10 ms).

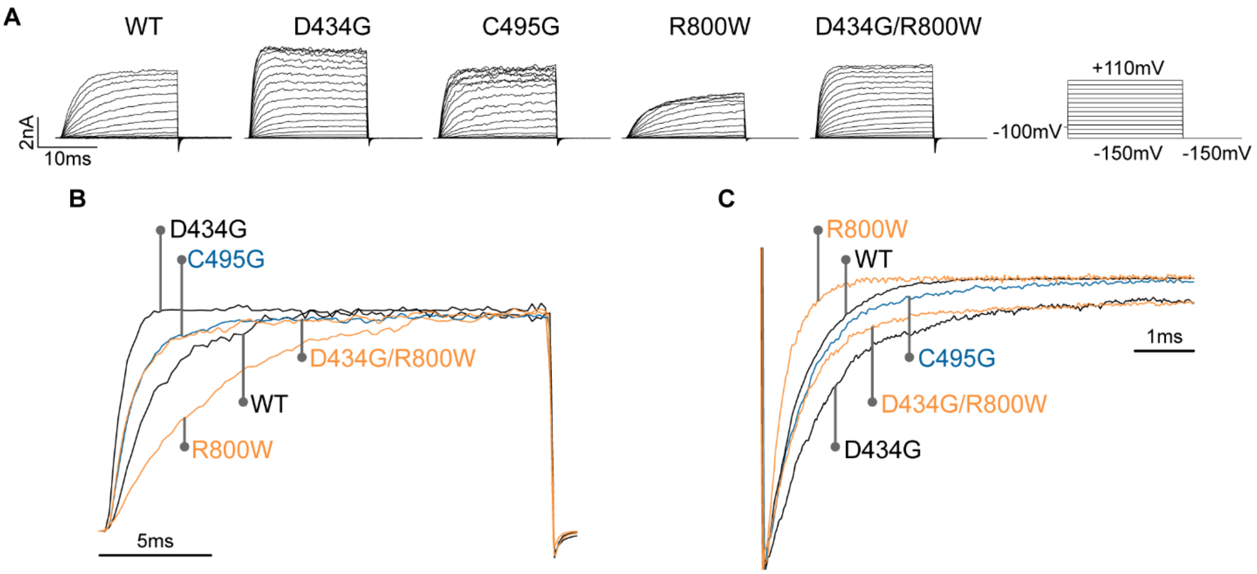

Figure S2. BK currents from WT and SNP-containing hBK channels in physiological K^+. **(A)** Representative macroscopic BK current traces in physiological K^+ at 10 μM Ca^{2+} from control wildtype (WT), D434G, C495G, R800W, and D434G/R800W-containing channels on the hBK_{VYR} splice variant background. Currents were evoked from a holding potential of -100 mV, stepping from -150 to +110 mV in +10 mV increments (20 ms), followed by a tail step to -150 mV (10 ms). **(B)** Representative current traces showing the difference in activation kinetics between WT, D434G, C495G, R800W, and D434G/R800W currents at +100 mV. Traces were scaled to the maximum steady-state current level for comparison. **(C)** Representative tail current traces demonstrating the differences in deactivation kinetics between WT, D434G, C495G, R800W, and D434G/R800W currents at the -100-mV tail step. Tail currents were evoked from an initial holding potential of -150 mV, followed by a voltage step to +200 mV for 20 ms and then 10 ms tail steps from -200 to -50 mV in +10 mV increments.

A138

```
                    S0-S1 linker
Human      HCGGKTKEA-------QKINNG-S-SQADGTLKPVDEKEEAVAAEVGWMTS 161
Monkey     HCGGKTKEA-------QKINNG-S-SQADGTLKPVDEKEEAVAAEVGWMTS 134
Pig        HCGGKTKEA-------QKINNG-A-SQADGTLKPVDEKEEVVAAEVGWMTS 135
Dog        HCGDKTKEA-------QKINNG-S-SQADGTLKPVDEKEEAVAAEVGWMTS 99
Cow        HCGGKTKEA-------QKINNG-S-SQADGTLKPVDEKEETVAAEVGWMTS 149
Rabbit     HCGGKAKEA-------QKINNG-S-SQADGTLKPVDEKEEAVAAEVGWMTS 162
Rat        HCGGKTKEA-------QKINNG-S-SQADGTLKPVDEKEEVVAAEVGWMTS 162
Mouse      HCGGKTKEA-------QKINNG-S-SQADGTLKPVDEKEEVVAAEVGWMTS 161
Chicken    HCGVKNKEA-------QKINGGGD-TQADGACKPTDEKEENVAAEVGWMTS 120
Zebrafish  HCNIKNKEA-------QKVNNPITI-QADGTTKTGNEKEEAPASEVGWMTS 115
Frog       HCGGKNKEA-------QKVVNVASSQVTDGDYKPTDDKEEVGVAEVGWMTE 129
Fly        RKEPDLGP------------------NDPKQKEQKASRNKQEFEGTFMTE 116
Worm       QRREKEFVEPIPAPEAVQINMNG-SKHAPSETDPF--LKQQEEKHLGWMTE 122
```

C495

```
             β sheet  flexible linker   α helix
Human      ESADACLIILANKYCADPDAEDASNIMRVISIKNYHPKIRII  522
Monkey     ESADACLIILANKYCADPDAEDASNIMRVISIKNYHPKIRII  495
Pig        ESADACLIILANKYCADPDAEDASNIMRVISIKNYHPKIRII  496
Dog        ESADACLIILANKYCDDPDAEDASNIMRVISIKNYHPKIRII  460
Cow        ESADACLIILANKYCADPDAEDASNIMRVISIKNYHPKIRII  510
Rabbit     ESADACLIILANKYCADPDAEDASNIMRVISIKNYHPKIRII  523
Rat        ESADACLIILANKYCADPDAEDASNIMRVISIKNYHPKIRII  523
Mouse      ESADACLIILANKYCADPDAEDASNIMRVISIKNYHPKIRII  522
Chicken    ESADACLIILANKYCADPDAEDASNIMRVISIKNYHPKIRII  481
Zebrafish  ESADACLIILANKYCADPDAEDASNIMRVISIKNYHPKIRII  476
Frog       ESADACLIILANKYCADPDAEDASNIMRVISIKNYHPKIRII  490
Fly        HEADACLVLANKYCQDPDAEDAANIMRVISIKNYSDDIRVI  471
Worm       GDADACLVLANKYSTNPDAEDAANIMRVISIKNYSSDIRVI  486
```

N599

```
                        β sheet           α helix
Human      EEDTWQKYYLEGVSNEMYTEYLSSAFVGLSFPTVCELCFVKLKLLMI  631
Monkey     EEDTWQKYYLEGVSNEMYTEYLSSAFVGLSFPTVCELCFVKLKLLMI  604
Pig        EEDTWQKYYLEGVSNEMYTEYLSSAFVGLSFPTVCELCFVKLKLLMI  605
Dog        EEDTWHKYYLEGVSNEMYTEYLSSAFVGLSFPTVCELCFVKLKLLMI  569
Cow        EEDTWQKYYLEGVSNEMYTEYLSSAFVGLSFPTVCELCFVKLKLLMI  619
Rabbit     EEDTWQKYYLEGVSNEMYTEYLSSAFVGLSFPTVCELCFVKLKLLMI  632
Rat        EEDTWQKYYLEGVSNEMYTEYLSSAFVGLSFPTVCELCFVKLKLLMI  632
Mouse      EEDTWQKYYLEGVSNEMYTEYLSSAFVGLSFPTVCELCFVKLKLLMI  631
Chicken    EEDTWQKYYLEGVANEMYTEYLSSAFVGLSFPAVCELVFAKLKLLMI  590
Zebrafish  EEDTWQKYYLEGVANEMYTEYLSSAFVGLSFPTVCELCYVKLKLLLI  585
Frog       EEDTWQKYYLEGVANEMYTEYLSSAFVGLSFPAVCELCFVKLKLLMI  599
Fly        DMQSWTNDYLRGTGMEMYTETLSPTFIGIPFAQATELCFSKLKLLLL  581
Worm       TTPDWLNLYLCGAGMEMYTDTLSHSFVGMTFPEAVDLLFNRLGLLLL  595
```

```
R800             β sheet           α helix                β sheet
Human      HVVVCIFGDVSSALIGLRNLVMPLRASNFHYHELKHIVFVGSIEYLK 887
Monkey     HVVVCIFGDVSSALIGLRNLVMPLRASNFHYHELKHIVFVGSIEYLK 802
Pig        HVVVCIFGDVSSALIGLRNLVMPLRASNFHYHELKHIVFVGSIEYLK 803
Dog        HVVVCIFGHVSSALIGLRNLVMPLRASNFHYHELKHIVFVGSIEYLK 767
Cow        HVVVCIFGDVSSALIGLRNLVMPLRASNFHYHELKHIVFVGSIEYLK 817
Rabbit     HVVVCIFGDVSSALIGLRNLVMPLRASNFHYHELKHIVFVGSIEYLK 830
Rat        HVVVCIFGDVSSALIGLRNLVMPLRASNFHYHELKHIVFVGSIEYLK 834
Mouse      HVVVCIFGDVSSALIGLRNLVMPLRASNFHYHELKHIVFVGSIEYLK 833
Chicken    HVVVCIFGDVKSALIGLRNLVMPLRASNFHYHELKHIVFVGSLEYLR 788
Zebrafish  HVVVCIFGDVTSALVGLRNLVMPLRASNFHYHELKPIVFVGSLDYLR 783
Frog       HVVVCIFGDMTSALIGVRNLVMPLRASNFHYHELKHIVFVGSLDYIK 798
Fly        HVVVCLFADPDSPLIGLRNLVMPLRASNFHYHELKHVVIVGSVDYIR 878
Worm       HVVVCLFADQDSPLIGLRNFIMPLRSSNFHYHELKHVVIVGDLEYLR 847
```

Figure S3. Evolutionary conservation of BK channel protein sequence for each SNP (yellow) and the surrounding residues. Structural features are annotated above each alignment and fully conserved residues across species are shown in grey. Alignments were produced using CLUSTAL Omega (v1.2.4)(Sievers et al., 2011) (available at www.uniprot.org/align/) between human (*homo sapiens,* uniprot accession: Q12791), monkey (*Macaca mulatta,* O18867), pig (*Sus scrofa,* O18866), dog (*Canis lupus familiaris,* Q28265), cow (*Bos Taurus,* Q28204), rabbit (*Oryctolagus cuniculus,* Q9BG98), rat (*Rattus norvegicus,* Q62976), mouse (*Mus musculus,* Q08460), chicken (*Gallus gallus,* Q8AYS8), zebrafish (*Danio rerio,* B7ZC96), frog (*Xenopus laevis,* Q90ZC7), fly (*Drosophila melanogaster,* Q03720), and worm (*Caenorhabditis elegans,* Q95V25) KCNMA1 protein sequences.

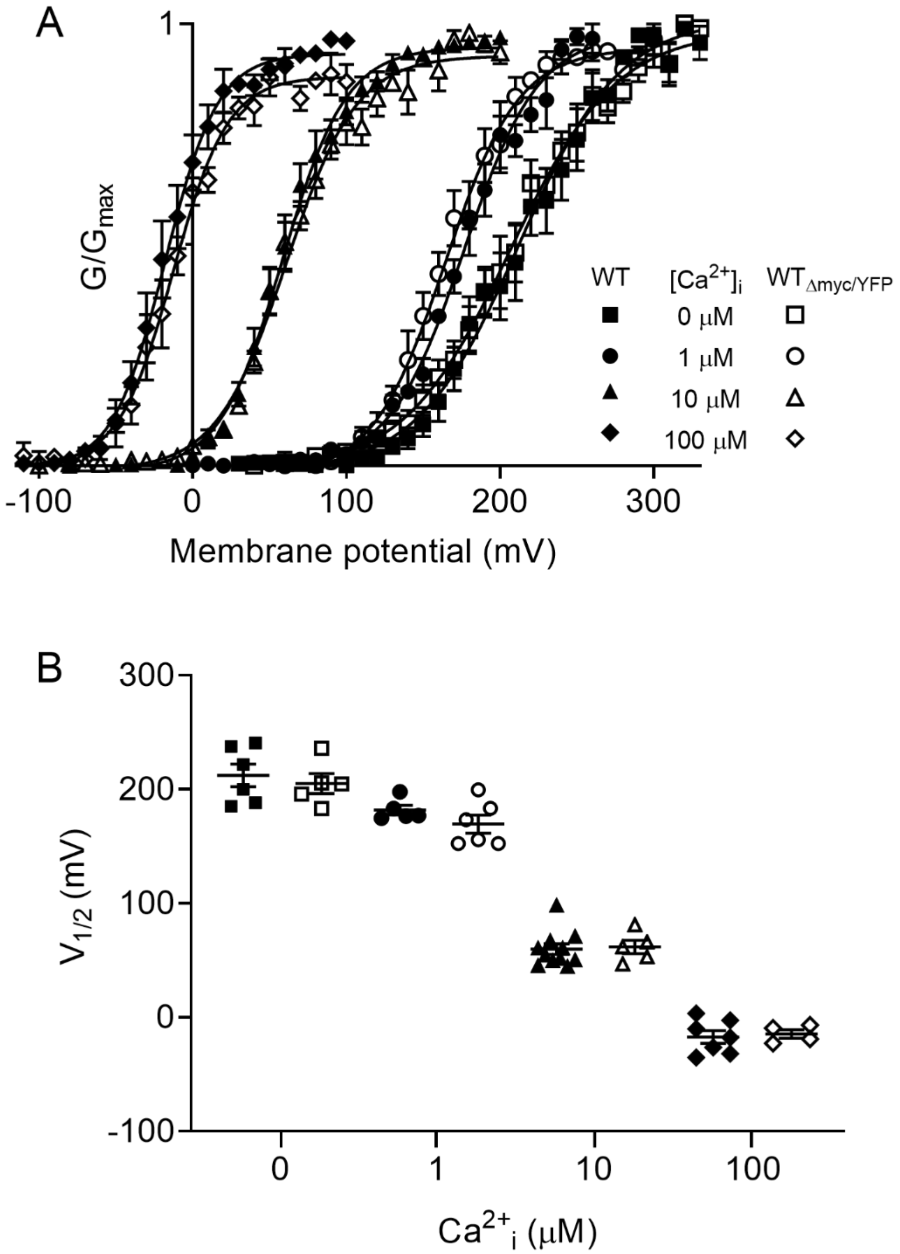

Figure S4. Comparison of WT channels with and without Myc and EYFP tags.
(A-B) Conductance-voltage relationships (A) and $V_{1/2}$ values (B) for macroscopic currents recorded from WT hBK_{VYR} channels with and without the N-terminal Myc tag and YFP tag ($WT_{\Delta myc/YFP}$) in symmetrical K^{+} at 0, 1, 10 and 100 μM $Ca^{2+}{}_{i}$. The N-terminal Myc tag (13 residues) and the EYFP tag (241 residues) inserted after residue 742 in the RCK2 domain were deleted by site-directed mutagenesis. BK currents were evoked using the voltage protocol stepping from -150 to +300 mV in +10 mV increments (20 ms) from a holding potential of -100 mV, followed by a tail step to -80 mV (10 ms) as in Figure 2. There were no significant differences in $V_{1/2}$ values between WT and $WT_{\Delta myc/YFP}$. $P > 0.05$, Student’s unpaired t-tests within each Ca^{2+} condition. N = 4–11 recordings per construct per condition.

Variant	SNP	RSID	Allele frequency	Validated	ClinVar
A138V	G>A	rs144215383	A=0.00041 (104/251436, GnomAD)<br>A=0.00202 (254/125568, TOPMED)<br>A=0.00054 (66/121324, ExAC)<br>A=0.0033 (256/78702, PAGE)<br>A=0.0015 (46/31398, GnomAD)<br>A=0.0015 (20/13006, GO-ESP)<br>A=0.002 (8/5008, 1000G)	Yes<br>(by cluster<br>&<br>frequency)	Likely benign
C495G	A>C	rs201243440	C=0.00003 (1/28004, dbSNP)	No	N/A
N599D	T>C	rs140520584	C=0.00001 (3/250970, GnomAD)<br>C=0.00002 (2/125568, TOPMED)<br>C=0.00002 (2/120876, ExAC)	Yes (by cluster & frequency)	N/A
R800W	G>A	rs199681253	A=0.00003 (1/28004, dbSNP)	No	N/A
D434G	T>C	rs137853333	C=0.00003 (1/31398, GnomAD)	Yes<br>(by cluster)	Pathogenic

Table S1. Allele frequency reports for SNP variants. Common variants are defined as a minor allele frequency of > 5% (0.05) (Strande et al., 2018). Only 5.6% of missense variants in the *KCNMA1* coding region are currently reported to have an allele frequency > 5% (ExAC database, Lek et al., 2016). All SNPs in this study classify as rare variants, defined as an allele frequency of < 1% (0.01) (MacArthur et al., 2014, Richards et al., 2015). A138V and N599S are validated by cluster, identified in multiple independent submissions to dbSNP, or by frequency, where the minor alleles were observed in at least two chromosomes (Sherry et al., 2001). C495G and R800W are identified as SNPs (Li et al., 2009) but are not yet validated via multiple allele submissions to dbSNP. A138V and D434G are reported in the ClinVar variant-phenotype database (Landrum et al., 2018) with predicted pathogenicity as indicated. Reported allele frequencies and information were compiled from the Database of Single Nucleotide Polymorphisms (dbSNP)(Sherry et al., 2001), comprised of the 1000 Genomes Project; ExAC, Exome Aggregation Consortium; GnomAD, Genome Aggregation Database; GO-ESP, Grand Opportunity Exome Sequencing Project; PAGE, Population Architecture using Genomics and Epidemiology; and TOPMED, Trans-Omics for Precision Medicine.

Supplemental References

Landrum, M.J., Lee, J.M., Benson, M., Brown, G.R., Chao, C., Chitipiralla, S. et al. (2018). ClinVar: improving access to variant interpretations and supporting evidence. *Nucleic Acids Research* 46, D1062-D1067.

Lek, M., Karczewski, K. J., Minikel, E. V., Samocha, K. E., Banks, E., Fennell, T. et al. (2016). Analysis of protein-coding genetic variation in 60,706 humans. *Nature* 536(7616), 285.

Li, R., Li, Y., Fang, X., Yang, H., Wang, J., Kristiansen, K. et al. (2009). SNP detection for massively parallel whole-genome resequencing. *Genome Res* 19, 1124-1132.

MacArthur, D. G., Manolio, T. A., Dimmock, D. P., Rehm, H. L., Shendure, J., Abecasis, G. R. et al. (2014). Guidelines for investigating causality of sequence variants in human disease. *Nature* 508(7497), 469.

Richards, S., Aziz, N., Bale, S., Bick, D., Das, S., Gastier-Foster, J. et al. (2015). Standards and guidelines for the interpretation of sequence variants: a joint consensus recommendation of the American College of Medical Genetics and Genomics and the Association for Molecular Pathology. *Genetics in Medicine*, 17(5), 405.

Sherry ST, Ward MH, Kholodov M, Baker J, Phan L, Smigielski EM. et al. (2001). dbSNP: the NCBI database of genetic variation. *Nucleic Acids Research* 29(1):308-11.

Sievers, F., Wilm, A., Dineen, D., Gibson, T.J., Karplus, K., Li, W., et al. (2011). Fast, scalable generation of high-quality protein multiple sequence alignments using Clustal Omega. *Molecular Systems Biology* 7**,** 539.

Strande, N. T., Brnich, S. E., Roman, T. S., and Berg, J. S. (2018). Navigating the nuances of clinical sequence variant interpretation in Mendelian disease. *Genetics in Medicine*, 20(9), 918.
